# Duration of O_2_ Exposure Determines Dominance of Fe^II^ vs CH_4_ Production in Tropical Forest Soils

**DOI:** 10.1021/acs.est.4c12329

**Published:** 2025-02-28

**Authors:** Diego Barcellos, Sherlynette Pérez Castro, Ashley Campbell, Jeffrey A Kimbrel, Steven Joseph Blazewicz, Jessica Wollard, Jennifer Pett-Ridge, Aaron Thompson

**Affiliations:** a Department of Crop and Soil Sciences, University of Georgia, Athens, Georgia 30605, United States; b Department of Environmental Sciences, Federal University of São Paulo (UNIFESP), Diadema, São Paulo 09913, Brazil; c Physical and Life Sciences Directorate, Lawrence Livermore National Laboratory, Livermore, California 94550, United States; d Adaptive Biotechnologies, Seattle, Washington 98109, United States; e Life & Environmental Sciences Department, University of California, Merced, California 95343, United States; f Innovative Genomics Institute, University of California, Berkeley, California 94720, United States

**Keywords:** tropical forest soils, redox oscillations, iron cycling, methane emissions, stable isotope probing

## Abstract

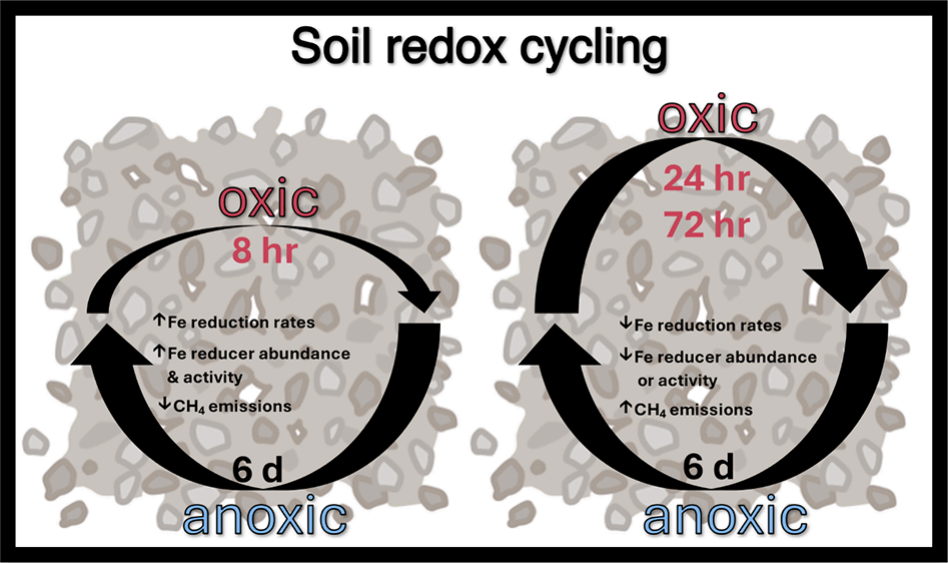

Temporal fluctuations in redox conditions influence the availability of Fe^III^ and greenhouse gas emissions in humid upland soils. However, the impact of fluctuation duration on biogeochemical processes remains unclear. We hypothesized that rates of Fe^III^ reduction and CH_4_ production are sensitive to the duration of soil oxygenation. To test this, surface soil from the Luquillo Forest, Puerto Rico, was subjected to fluctuating redox conditions with an anoxic interval of 6 days followed by oxic intervals of either 8, 24, or 72 h. Shorter oxic intervals enhanced Fe reduction, while longer oxic intervals enhanced CH_4_ emissions. As O_2_ exposure decreased from 72 to 8 h, Fe reduction rates increased from 0.12 ± 0.02 to 0.26 ± 0.05 mmol kg^–1^ h^–1^, whereas cumulative CH_4_ decreased from 44.0 ± 4.7 to 12.7 ± 4.6 μmol kg^–1^. ^13^C-amino acid spikes were preferentially incorporated into the DNA of iron reducers (*Anaeromyxobacter* sp.) in the shorter oxic treatment (8 h vs 24 h), suggesting that Fe reducers are less inhibited by shorter periods of oxidation. Conversely, longer oxygen pulses appear to suppress Fe reducers more than methanogens, leading to increased CH_4_ emissions. These findings highlight the role of the redox oscillation length in modulating biogeochemical processes and greenhouse gas emissions in soils.

## Abstract 2

The length of O_2_ exposure determines anaerobic rates of soil Fe^III^ reduction and CH_4_ production in upland soils under redox oscillations.

## Introduction

Redox variability is ever present in soils^1−3^ and drives critical biogeochemical transformations of iron (Fe), methane (CH_4_), and other compounds.^4−7^ This is most evident in the spatial redox heterogeneity that emerges within microsites and along flow paths and can yield vastly different redox conditions of soil locales separated by a cm or less.^8−10^ Oxygen (O_2_) depletion commonly manifests within aggregates, along the margins of preferential flow paths, within small pores or other features that restrict the water flow in the presence of organic matter.^11−14^ Common visual expressions of spatial redox heterogeneity in soils include iron mottling, concretions, and Liesegang bands.^15−18^ Temporal redox variability is also common and emerges within individual microsites or in bulk soil pores due to the shifting water content, carbon availability, and O_2_ depletion.^19−21^ While spatial redox heterogeneity manifests as distinct redox-static biogeochemical niches, temporal redox heterogeneity forces direct competition between microbial groups and thus generates niches where soil taxa must tolerate redox conditions that are dynamic.^22−24^ Pett-Ridge et al.^25,26^ showed that specific microbial communities maintain adaptation to shifting redox conditions and, in some tropical forest soils, have adapted to specific redox fluctuation periodicity, including oscillations as short as every 4 days. Altering the periodicity in these soils shifted the microbial community and its function.^26,27^

Currently, redox variability is represented in global biogeochemical models via discrete redox-sensitive processes that are turned on or off based on soil moisture.^4,28,29^ In this manner, rates of CH_4_ or CO_2_ production (for instance) are scaled to the time under anoxic or oxic conditions.^20,30,31^ This is a valid approach only if redox status has only two conditions and if soil moisture can be taken as a reasonable proxy for redox status, such that the pattern of redox changes does not matter, only whether the system was oxic or anoxic.^32^ However, we argue that the shape of redox fluctuation patterns does matter, at least for some processes and for some fluctuation parameters. Three redox fluctuation parameters can reasonably define the pattern of redox fluctuations: periodicity (the recurrence rate of low redox events), amplitude (the rates of O_2_ introduction or consumption), and duration (the length of time that any low or high redox condition persists; Figure S1).^19,31,33,34^

Prior exposure to anoxic conditions is known to impact anaerobic biogeochemical processes by conditioning indigenous anaerobic communities.^35−37^ However, if the period of time between anoxic events is too long, then anaerobic communities may lose this conditioning, or aerobes may become relatively more dominant; conversely, too short an anoxic event and certain anoxic processes with less thermodynamic yield (e.g., CH_4_ generation) may never develop.^38−40^ Most anaerobic processes also depend on electron acceptors (e.g., NO_3_^–^, Mn^IV^, Fe^III^, and SO_4_^2–^) that can be renewed biotically or abiotically by a pulse of O_2_, although the kinetics of oxidation varies.^41−43^ Furthermore, organic compounds in soils with periodic oxic conditions tend to have a higher average nominal oxidation state of carbon (NOSC) value,^44^ which can increase the concentration of organic matter electron donors that are more thermodynamically favorable for anaerobic processes.^45,46^

Periodicity and the duration of O_2_ reintroduction are clearly important regulators of biogeochemical processes, especially at the extremes of very short fluctuations or the difference between a fluctuating system and an essentially permanently oxic or anoxic system.^9,20,28,47^ For instance, while redox fluctuations generally increase Fe reduction rates relative to nonredox fluctuating systems,^33,34^ variations in periodicity do not appear to impact these rates unless the frequency becomes very rapid.^19^ In the case of Fe reduction, reoxidation of Fe^2+^ generates fresh electron-accepting Fe^III^ phases that are preferentially reduced over bulk Fe^III^ (i.e., rapidly reducible Fe^III^), with faster oxidation rates (amplitude) producing more rapidly reducible Fe^III^ minerals than slower oxidation events.^31^ If the availability of electron acceptors is limiting, then maximum Fe reduction rates should occur at short redox oscillation frequencies, as Calabrese et al.^28^ has shown in a theoretical paper predicting maximal Fe reduction rates as a function of the redox dynamics using the frequency and mean depth of rainfall events.^48^ However, this does not account for the timescales of microbial growth and activation, which constrain oxygen consumption and biogeochemical processes in redox dynamic systems. In some humid upland soils, redox conditions oscillate on timescales that are shorter than organisms can respond via population growth.^20,23,25^ Under these conditions, the ability of microbes to adjust their metabolism and tolerate changes in oxygen availability may be important strategies.^49,50^ At the pedon scale, this would allow O_2_ respiring taxa to coexist with fermenters and a wide variety of anaerobes/facultative taxa that use terminal electron acceptors other than O_2_.

We expect that the integrated biogeochemical responses to dynamic redox conditions will depend considerably on how various processes and their abiotic and biotic drivers are alternately constrained or enhanced. Since very short redox fluctuations should stimulate Fe reduction,^19,28^ we sought to evaluate the role of O_2_ exposure length on Fe reduction and competing anaerobic processes. Here, we focus on the production of CH_4_, which can occur through a combination of fermentation and anaerobic microbial respiration^51^ and may be suppressed when Fe reducers outcompete methanogens for acetate and hydrogen substrates.^37,52^ Conversely, methanotrophs can participate in Fe^III^ oxide reduction via CH_4_ oxidation under anoxic conditions;^35,53,54^ this may be an important sink for CH_4_ in soils.^55^ We hypothesized that during redox fluctuations, soils exposed to brief oxic intervals (τ_oxic_) would exhibit higher Fe reduction rates during subsequent anoxic intervals (τ_anoxic_) than those exposed to longer oxic intervals and that these higher Fe reduction rates would suppress CH_4_ emissions. We tested this by exposing a redox fluctuating soil to variable lengths of oxic exposure (maintaining similar lengths of anoxia) while monitoring Fe^II^ concentrations, CO_2_ and CH_4_ efflux, Fe mineral composition, and soil microbial community composition.

## Methods

### Site and Sample Characterization

Five soil cores were collected from 0–10 cm depth in a valley location at the Bisley Research Watershed in the Luquillo Experimental Forest (LEF), Puerto Rico (Luquillo Critical Zone Observatory, LCZO). The samples were collected under field-moist conditions, placed in plastic bags, stored under oxic conditions in a cooler at ambient temperature, and immediately shipped to the University of Georgia within 24 h of sampling. The field-moist samples were then carefully homogenized and sieved (2 mm) under anoxic conditions in a 95%:5% (N_2_:H_2_) glovebox^56^ (Figure S2). The initial soil moisture content of the fresh soil was 77% (0.77 g of water per g of dry soil). Soils from the Bisley watershed are predominantly ultisols (Typic Haplohumults) formed from volcanic parent material, weakly acidic, and mineralogically composed of quartz, kaolinite, chlorite, and goethite.^57^ The soil redox oscillates on timescales of several days.^22^ Total Fe and Al were determined by ICP-MS following a Li-metaborate fusion. Standard short-range-ordered (SRO) Fe and Al phases were obtained by citrate/ascorbate extraction (0.2 M sodium citrate/0.05 M ascorbic acid) and analyzed by ICP-MS. The native soil (prior to incubation) contained 943 ± 4 and 439 ± 7 mmol kg^–1^ soil of total Fe and SRO Fe^III^, respectively (Table S1).

### Redox Oscillations and Iron Reduction

We subjected natural soils in suspensions (1:10 soil:solution ratio) to three different redox oscillation treatments for up to 47 days. Soil slurries (suspensions) were constantly mixed on an orbital shaker (250 rpm) to decrease soil heterogeneity and force microbial interactions, essentially magnifying the competition that might occur within a single microsite.^9^ Our experimental design was similar to that described by Barcellos et al.,^19^ but with fresh, field-moist soils instead of air-dried soils (to better capture the ambient microbial community dynamics). The soil slurries were buffered to maintain the natural soil pH (5.5) with MES (2-*N*-morpholino-ethanesulfonic acid) with KCl as a background electrolyte. Each treatment contained triplicate reactors (Nalgene polypropylene narrow-mouth Erlenmeyer flask), which contained 4.5 g (dry-weight equivalent) of soil in a 2 mM KCl + 10 mM MES that buffered solution at pH 5.5 ± 0.2 across the duration of the experiment, with a 45 g final suspension mass. Soil slurries were placed in a 95%:5%:0% (N_2_:H_2_:O_2_) glovebox (Coy anaerobic chamber) for the anoxic condition and were exposed to laboratory room air (∼21% O_2_) for the oxic condition, both constantly shaking on an orbital shaker at 250 rpm and in the dark. Fe^II^ was measured every 8–72 h in 0.5 M HCl extraction, which combines the aqueous and adsorbed Fe^II^. Based on previous studies,^31,34,58^ most of the Fe^II^ involved in the redox cycling of soils is surface-adsorbed, with only a small portion present in the aqueous phase; thus, we present HCl-extractable Fe^II^ values, which contain both adsorbed and aqueous Fe^II^. We accomplished the extraction by withdrawing 0.5 mL of the suspension from the same vessel using wide orifice pipet tips (to avoid soil particle size exclusion and keeping the same soil:solution ratio), adding 0.5 M HCl, shaking for 2 h, centrifuging at 11,000 RCF (relative centrifugal force) for 10 min, and taking the supernatant for analysis.^56,59^ Concentrations of Fe^II^ after ferrozine colorimetric analysis were obtained from 562 nm (and 500 nm) in a spectrophotometer.^60^

In parallel reactors, dissolved O_2_ (DO) was monitored through a single redox oscillation cycle (undergoing oxic and anoxic conditions) in triplicate reactors using a Hach (USA) DO meter. Within 1 h after exposing anoxic soil slurries to oxic conditions, DO increased to >7.0 mg L^–1^ (>84.7%). Likewise, for soil slurries that were previously exposed to oxic conditions over 24 h, we observed that DO decreased from 9.90 to 0.24 mg L^–1^ (∼100 to 2.4%) within 2 h and reached 0.06 mg L^–1^ (0.73%) after 24 h of anoxia (Table S2).

We aimed to test the influence of O_2_ exposure on Fe reduction rates and implications on CO_2_ and CH_4_ emissions by changing the time that soils would be exposed to O_2_ (τ_oxic_) from 72, 24, and 8 h coupled with a long anoxic period (τ_anoxic_) of 144 h (6 days) (Table 1). We started our experiment by preconditioning all reactors to three sequential oscillation periods of 6-day anoxic and 1-day oxic, in order to acclimate the soil’s microbial communities to repetitive identical shifts in redox conditions. Thus, after the preconditioning period (at 480 h), we split the reactors into three treatments, undergoing three consecutive redox cycles as follows: Ox-72 with 144 h anoxic + 72 h oxic, Ox-24 with 144 h anoxic + 24 h oxic, and Ox-8 with 144 h anoxic + 8 h oxic (Table 1). Control treatments with either constant anoxic or oxic conditions were also included (*n* = 3).

**Table 1:**
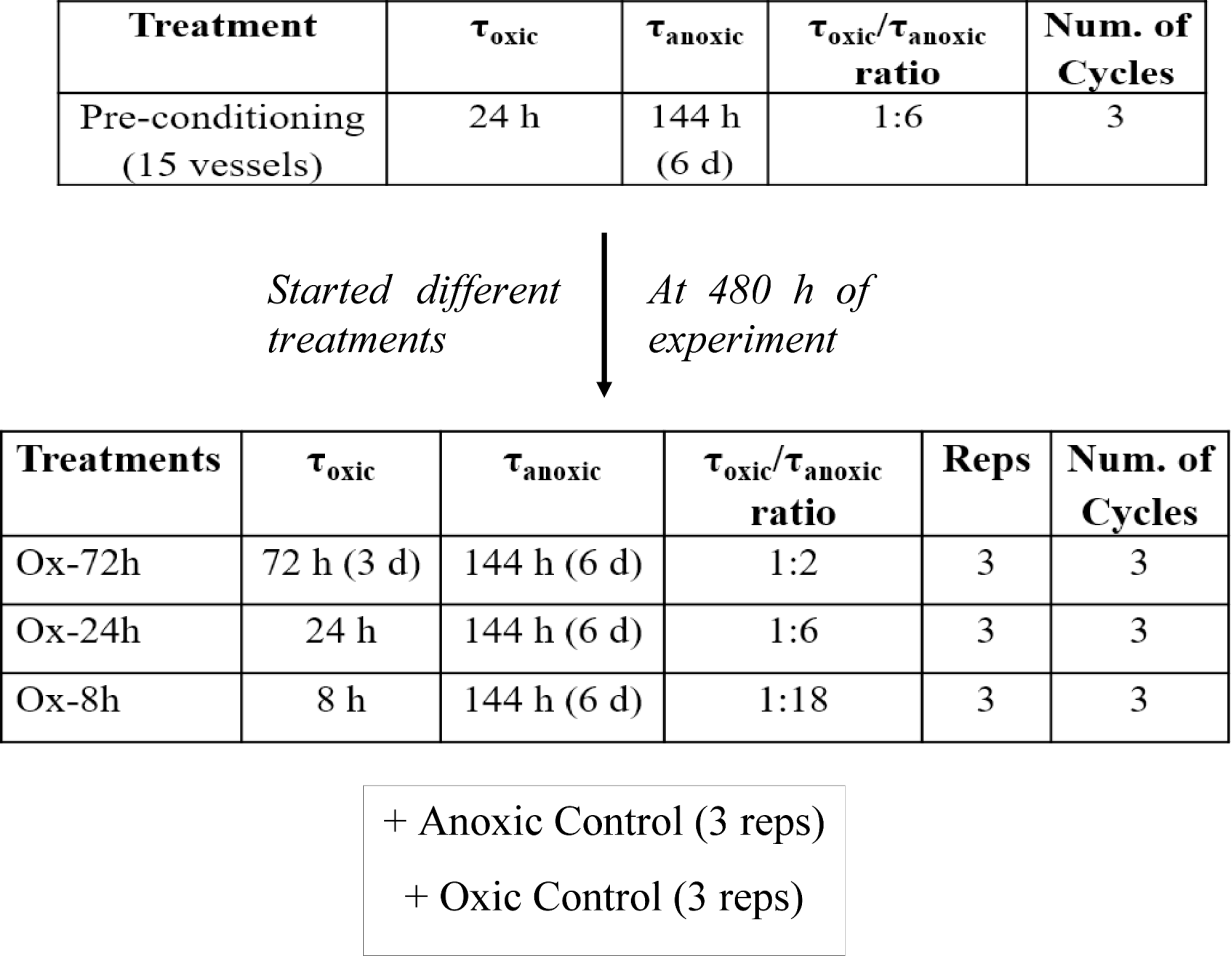
Treatments for Different Oscillation Periods and τ_oxic_ or τ_anoxic_ Durations in Hours (and Days) from a Tropical Forest Soil Incubation Experiment (Luquillo Experimental Forest, Puerto Rico), with Different Redox Oscillation Treatments

### Trace Gases and Carbon Analyses

Fluxes of CO_2_ (in mmol kg^–1^ of soil h^–1^) and CH_4_ (in μmol kg^–1^ of soil h^–1^) were measured at approximately the beginning, middle, and end of each redox cycle. For each gas flux measurement, we capped the triplicate reactors with rubber septa and sampled the headspace gas at 0, 10, and 30 min with gastight syringes and stored in pre-evacuated 3 mL glass vials. Samples were analyzed in a gas chromatograph (Shimadzu GC-14A, Japan) using a flame ionization detector (FID) and an electron capture detector (ECD). Nitrogen (280 kPa) was used as the carrier gas, and the flow in the column was 24.3 mL min^–1^. Measurements of CO_2_ and CH_4_ were used to calculate both instantaneous fluxes (in mmol kg^–1^ of soil h^–1^ and μmol kg^–1^ of soil h^–1^, respectively) and estimated cumulative fluxes (in mmol kg^–1^ of soil and μmol kg^–1^ of soil, respectively), calculated by multiplying the instantaneous flux by all hours prior to the current measurement and after that last measurement. We note that these cumulative flux estimates do not capture gas loss between measurements.

Samples for total carbon and nitrogen were analyzed via combustion in a CHN Carlo Erba elemental analyzer. The native soil (no treatment added) had 37.4 mg g^–1^ of total C and 2.2 mg g^–1^ of total N (solid phase). The MES buffer added another 7.5 mg of C, comprising 14% of carbon in each reactor, which made for 44.9 mg g^–1^ of total C at the start of the experiment. Dissolved organic carbon (DOC) from the aqueous phase (supernatant after centrifugation) of the soil slurry at the end of redox oscillation for each treatment was measured in a Shimadzu 5050 TOC.

### Mössbauer Spectroscopy

Detailed Fe speciation was determined by Mössbauer spectroscopy at the temperatures of 50, 35, 25, 13, and 5 K. We collected triplicate soil samples at the end of the last (third) oxic interval for the treatments Ox-72, Ox-24, and Ox-8 and for the common soil used in all treatments at the beginning of the experiment (initial soil). We pooled together the triplicate oxic samples to form one soil sample, placed those samples in a ring that was covered with Kapton tape to avoid gas diffusion, and immediately froze the sample in a −20 °C freezer. The samples were placed in our Mössbauer spectrometer’s cryostat (precooled to below 140 K), operating with a He atmosphere to prevent the oxidation of any Fe^II^ by oxygen. The Mössbauer spectra were recorded in transmission mode with a He-cooled cryostat with variable temperature (Janis Research Co.) and a channel detector (1024). Detailed information for the Mössbauer spectra modeling and fitting parameters (Figures S3–S6 and Tables S3–S6) are provided in the Supporting Information.

### Microbial Analyses

To identify Fe reducers, methanogens, and the overall soil microbial community composition, DNA was extracted from soil slurries at the beginning of the experiment (*n* = 3) and at the end of the last anoxic interval for Ox-72 (*n* = 2), Ox-24 (*n* = 2), and Ox-8 (*n* = 2) using an in-house phenol-chloroform extraction (Supporting Information). The 16S rRNA V4 region was amplified using the primers 515F and 806R targeting bacteria and archaea.^61,62^ Amplicons were sequenced on a MiSeq using Illumina’s v3 500-cycle (paired-end) reagent kit at the Argonne National Laboratory Next Generation Sequencing Core Facility. Raw sequences were processed, and amplicon sequence variants (ASV) were generated using Qiime v1.9.1 (Supporting Information). Fe reducers and methanogens were identified from species documented in the literature (see Section 2 of the Supporting Information). The community composition was obtained using ASV relative abundances.

To determine if the Fe reducers and methanogens detected in the 16S rRNA gene survey were metabolically active, 44 mg of isotopically labeled amino acids (AA) (99 atm% ^13^C and ^15^N; Sigma) was added to slurries for the Ox-08 (*n* = 3) and Ox-24 (*n* = 3) treatments, 24 h before the last oxic interval. The slurries were incubated for one more oxic and anoxic cycle. After 1 week of incubation, 11 mL (∼1 g of soil) was withdrawn, flash-frozen, and stored at −80 °C for subsequent microbial community analysis. Density-based stable isotope probing (SIP) fractionation was then performed using the high-throughput SIP pipeline^63^ at the Lawrence Livermore National Laboratory (Supporting Information). 16S rRNA gene sequences were amplified with the 515F and 806R PCR primers, and the resulting amplicons were paired-end sequenced on a MiSeq sequencer at the Lawrence Livermore National Laboratory (Supporting Information). Raw sequences were processed, and ASVs were generated using DADA2 v1.28^64^ (Supporting Information). The community composition was obtained using ASV relative abundances. To identify active Fe reducers, we used the conservative approach of only sequencing fractions with a density of >1.7525 g mL^–1^. The buoyant density of natural abundance DNA in CsCl solution increases with an increasing genome GC content, and the highest GC content reported in bacteria/archaea is 75%. This GC content can be used to estimate the highest average buoyant density possible for natural abundance DNA using the formula: buoyant density = (0.098 × 0.75) + 1.66 = 1.7335 g mL^–1^. Since the DNA of a single population follows a Gaussian distribution in the CsCl density gradient, we estimated the range of this distribution using pure culture SIP samples in our specific density gradient profiles. This range spans ±0.019 g mL^–1^, resulting in an upper limit of 1.7525 g mL^–1^ (1.7335 + 0.019) for natural abundance DNA. Consequently, we concentrated our sequencing efforts on DNA with higher buoyant density exceeding this cutoff to ensure that we identified only those taxa whose DNA density increased due to isotope incorporation during replication.

### Analyses of Metabolites (Acetate) by NMR

We collected the aqueous phase from the reactors (supernatant after centrifugation) at the end of the last (third) oxic interval for the treatments Ox-72, Ox-24, Ox-8, and preconditioning, to perform metabolite analyses (acetate) by nuclear magnetic resonance spectroscopy (NMR). Details for the analyses are provided in Section 3 of the Supporting Information.

### Statistical Analyses

To compare the effect of the different redox oscillation treatments on Fe^II^ concentrations and cumulative CO_2_ and CH_4_ fluxes, we performed ANOVA analysis using a Kenward–Roger approximation and parametric bootstrap function for linear mixed models, using the lmer function from the lme4 package in R.^65,66^ To correlate the effect of preceding τ_oxic_ on anaerobiosis of Fe and C, we computed linear regressions individually for each of the treatments comparing two of these variables at a time (Fe^II^ and CH_4_), under anoxic conditions only, using the lm function from the lme4 package in R. We further conducted a one-way ANOVA to test for differences in acetate concentrations among the treatments, for the soil samples collected in the last (third) oxic interval. Statistical Analysis of Metagenomic Profiles (STAMP) was used to identify significant differences in taxonomic groups among treatments.^67^

## Results and Discussion

### Ferrous Iron Dynamics and Iron Reduction Rates

**Figure 1:**
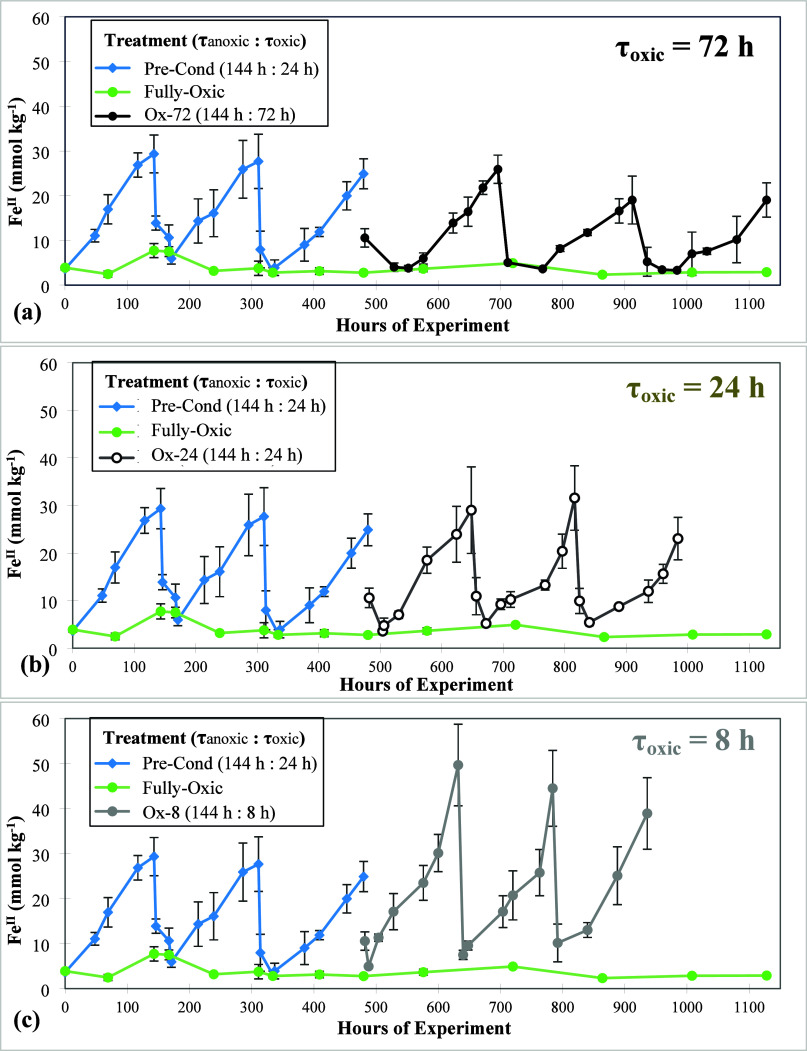
Soil Fe^II^ dynamics for soils from the Luquillo Experimental Forest (Puerto Rico) incubated with multiple headspace redox treatments (mmol of Fe^II^ per kg of dry-weight equivalent soil: mean ± 1 standard deviation), including a preconditioning period (τ_oxic_ = 24 h), a fully oxic treatment, and three treatments with decreasing τ_oxic_ of 72, 24, and 8 h (a, b, and c, respectively). In the oxic control treatment (nonfluctuating), Fe^II^ concentrations remained steady (3.8 ± 0.5 mmol kg^–1^) throughout the experiment. Data from a static anoxic control incubation are presented in Figure S7.

We cycled all treatments through three preconditioning redox cycles (6-day reduction followed by 24 h of oxidation; Table 1) to verify that all replicates were behaving similarly and exhibiting significant increases in Fe^II^ during the anoxic intervals and sharp drops in Fe^II^ during the oxic periods (Figure 1); this preconditioning also removed any “start-up” effects of the experiment. After the preconditioning cycles, we split the treatments for an additional three redox cycles so that three replicates each had either an 8, 24, or 72 h exposure to O_2_ followed by again a similar 6-day anoxia (Table 1, Figure 1, and Figure S7). All replicates continued to behave as expected, with Fe^II^ increasing during anoxic periods, followed by sharp drops when O_2_ was reintroduced;^19,24^ Fe reduction rates and peak Fe^II^ concentrations decreased slightly but remained statistically similar (*p* > 0.05) within a given treatment from the first to the third experimental redox cycle.

**Figure 2:**
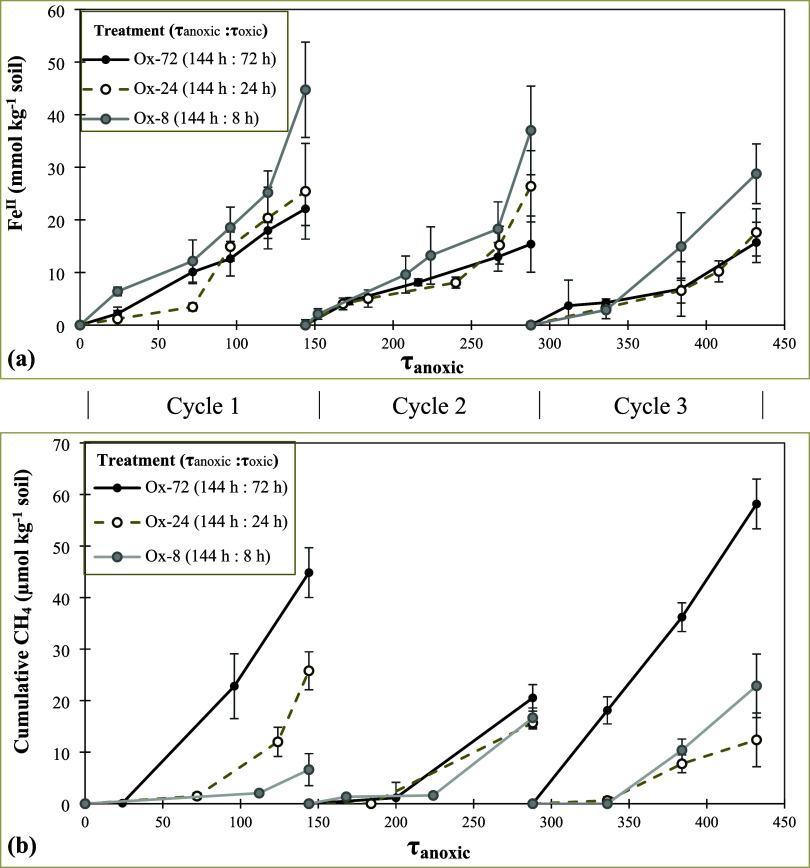
(a) Fe^II^ concentrations normalized to the initial concentration at each cycle (mean ± 1 standard deviation) over anoxic conditions (τ_anoxic_) only, for the treatments with decreasing τ_oxic_ of 72, 24, and 8 h; (b) cumulative CH_4_ normalized to the initial concentration at each cycle (mean ± 1 standard deviation) over anoxic conditions (τ_anoxic_) only, for the treatments with decreasing τ_oxic_ of 72, 24, and 8 h. Soils from the Luquillo Experimental Forest (Puerto Rico). τ_anoxic_ is the cumulative number of hours in the anoxic condition.

**Figure 3:**
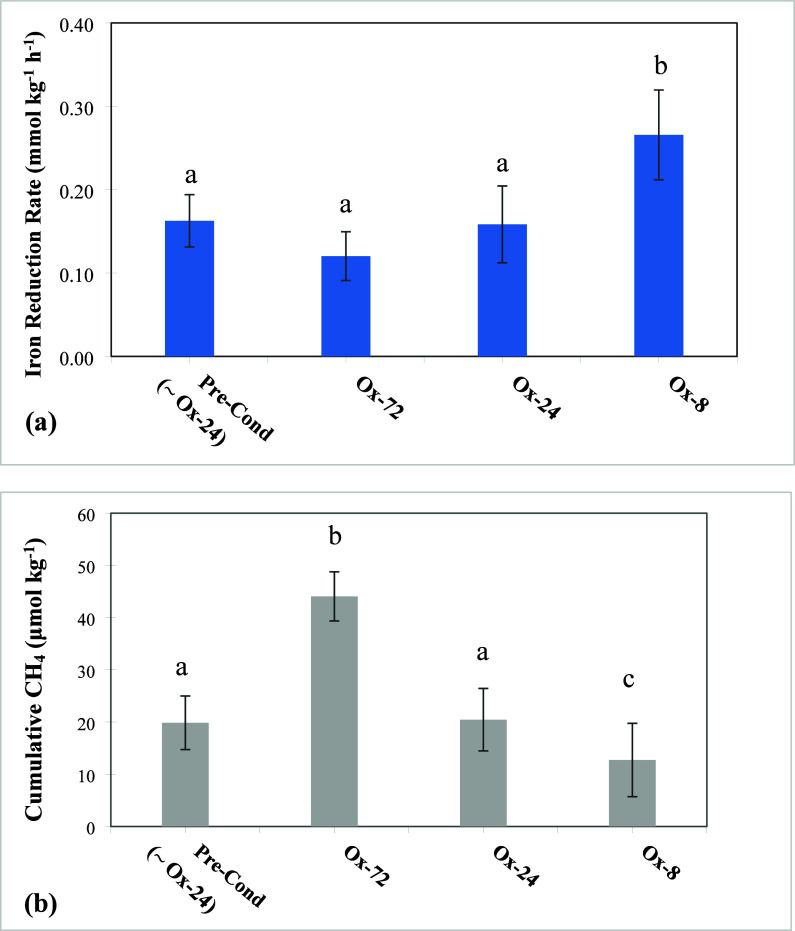
(a) Average Fe reduction rates with *n* = 3 redox cycles for the preconditioning and treatments with τ_oxic_ of 72, 24, and 8 h. (b) Cumulative CH_4_ during τ_anoxic_ for each treatment. Lowercase letters in parentheses (a and b) indicate significant differences at the 5% probability level. The error bars indicate a ±1 standard deviation. Soils from the Luquillo Experimental Forest (Puerto Rico). Summary of results: alterations in Fe^II^ and CH_4_ during anoxic conditions (τ_anoxic_), with changes in the preceding τ_oxic_ for the different treatments.

We found that anoxic Fe^II^ production differed depending on the length of oxic exposure. Incubations with the shortest O_2_ exposure (Ox-8 treatment) had both greater Fe^II^ concentrations (Figure 2a) and higher Fe reduction rates (0.26 ± 0.05 mmol kg^–1^ h^–1^; Figure 3a) relative to the Ox-24 and Ox-72 treatments (Figure 2a). These Ox-24 treatment had similar Fe reduction rates (0.16 ± 0.03 mmol kg^–1^ h^–1^) to the preconditioning period (which had identical τ_oxic_ and τ_anoxic_), while rates in the Ox-72 treatment were slightly lower (0.12 ± 0.02 mmol kg^–1^ h^–1^), but the difference was not significant (Figure 3a).

### Shorter O_2_ Perturbations Stimulated Faster Fe Reduction Rates

Short pulses of O_2_ (τ_oxic_) evidently stimulate higher anoxic Fe reduction rates during subsequent periods of anoxia. We discuss potential explanations for this by considering in turn the factors governing soil Fe reduction rates, principally: the availability of Fe^III^ electron acceptors, the availability of labile carbon substrates (electron donors), and the activities of microbial Fe reducers.^22,49,68,69^ Higher Fe reduction rates following a shorter O_2_ exposure time could be explained by a greater abundance of electron acceptors, more available electron donors, and a more active Fe reducer population in those treatments.

**Figure 4:**
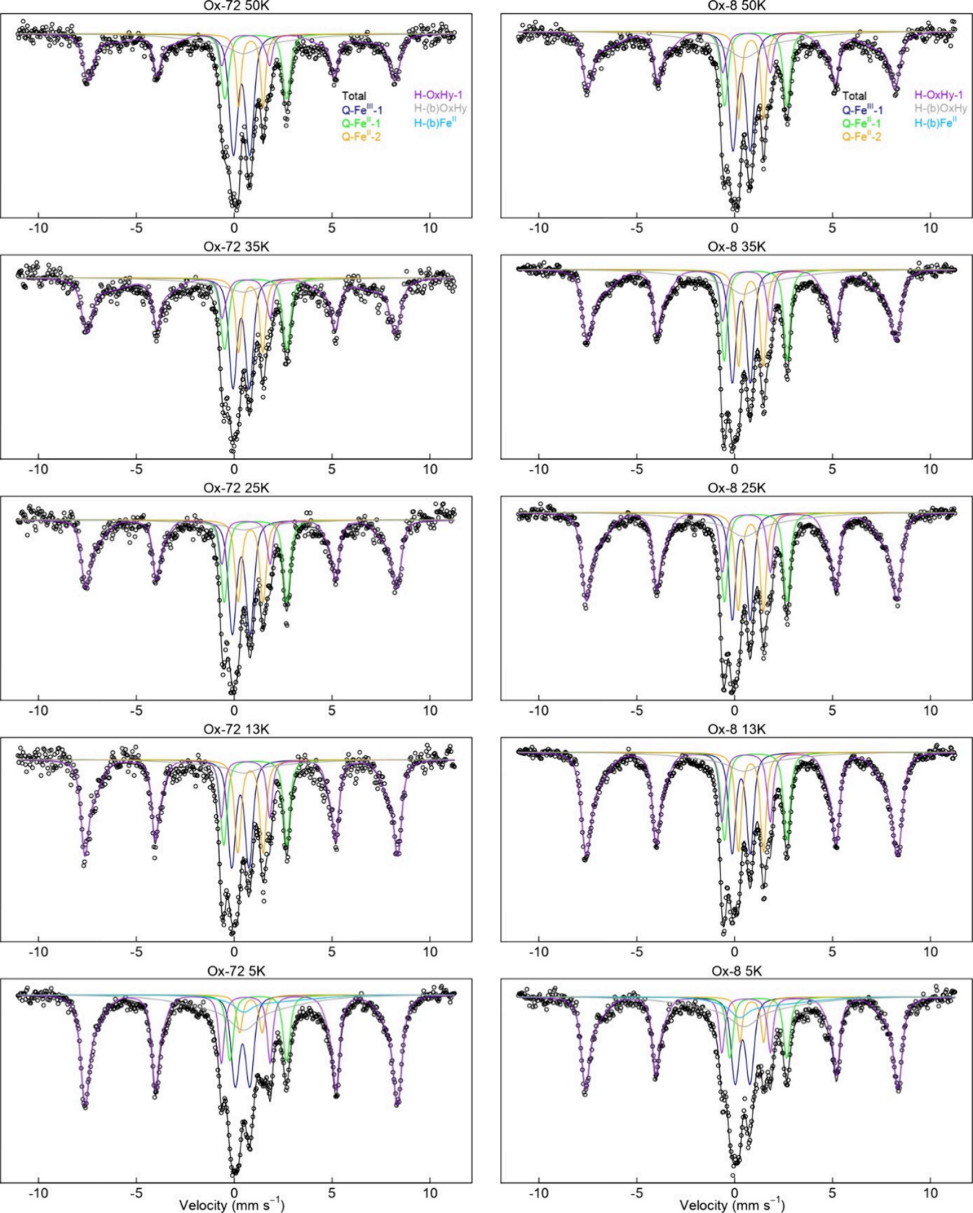
Mössbauer spectra (50, 35, 25, 13, and 5 K) for the Luquillo Experimental Forest soils (Puerto Rico) collected at the end of the last (third) oxic interval, for the redox oscillation treatments Ox-72 and Ox-8. For each spectrum, the black line corresponds to the total calculated fit, through the discrete data points. The resolved spectral components and assignments are (1) Q-Fe^III^-1, the deep central doublet (blue line) corresponding to Fe^III^ in aluminosilicates or organic matter; (2) Q-Fe^II^-1, the wider ferrous doublet corresponding to adsorbed Fe^II^ or Fe^II^ in clays or organic matter (green line); (3) Q-Fe^II^-2, the narrow ferrous doublet corresponding to ilmenite (brown line); (4) HFD-OxHy-1, the dominant sextet (purple line) corresponding to Fe^III^-oxyhydroxides that are magnetically ordered; (5) HFD-(b)OxHy, the collapsed “sextet” corresponding to Fe^III^ oxyhyroxides near their blocking temperature; and (6) H-(b)Fe^II^, the partially magnetically ordered Fe^II^ phase. Detailed fitting parameters are provided in the Supporting Information (Tables S3 and S6).

To assess differences in the availability of Fe^III^ electron acceptors, we analyzed solid-phase samples by Mössbauer spectroscopy at the end of the last (third) oxic interval for the contrasting treatments Ox-8 and Ox-72. Mössbauer spectroscopy is highly sensitive to the crystallinity of Fe oxide phases when run across a temperature gradient, with less crystalline phases, which are typically more available for Fe^III^ reduction, requiring a lower collection temperature to magnetically order into a Mössbauer sextet.^7^ We found that the Mössbauer sextet abundance was similar for both the Ox-8 and Ox-72 samples at 50, 35, and 5 K, with slightly higher sextet abundance in the Ox-8 samples (46.4 ± 1.1 and 51.6 ± 1.0%) than the Ox-72 (41.7 ± 2.4 and 46.9 ± 2.4%) samples at 25 and 13 K, respectively (Figure 4, Figures S3–S6, and Tables S3–S6). One could interpret this as evidence that the Ox-8 samples had higher crystallinity (and thus likely less availability for Fe reduction) than the Ox-72 samples. However, another measure of crystallinity is the hyperfine field strength of the sextet; the Ox-8 sextets at 25 and 13 K are more skewed toward lower hyperfine field strengths (Bhf 47.5 and 47.9, respectively) than the Ox-72 sextets (Bhf 47.9 and 48.4, respectively), which suggests that the portions of Fe phases in the Ox-8 samples that order at 25 and 13K, while more abundant than the in Ox-72 samples, are comparatively less crystalline. In all cases, these differences are minor and much less pronounced than changes in both treatments relative to the initial soil (Figures S3–S6 and Tables S3–S6), or changes reported previously in response to redox fluctuations.^60,70^ This suggests that no significant differences in SRO-mineral crystallinity exist following the oxidation events. Furthermore, while it is well-understood that pO_2_ (as well as Fe^II^ oxidation rates^7,31,71^) impacts the formation and crystallinity of incipient Fe^III^ minerals, all of our treatments were exposed to similar pO_2_ (∼21% O_2_). The length of O_2_ exposure (8–72 h) could feasibly generate different amounts of crystal ripening as some find in laboratory syntheses at high temperatures^72^ and under acidic conditions,^73^ but our Mössbauer data suggest that this does not happen in our experiment.

We also tested for differences in labile organic matter (electron donors) by measuring water-extractable DOC present at the beginning of the final anoxic interval. Total DOC (corrected for the abundance of the MES organic buffer) was statistically similar (*p* > 0.05) for the Ox-72 and Ox-8 soils (155 ± 13 and 170 ± 21 mg L^–1^, respectively). We also used nuclear magnetic resonance spectroscopy (NMR) to evaluate the volatile fatty acids (VFAs) in the samples and found that acetate concentrations were not statistically different (*p* > 0.05) between the precondition, Ox-8, Ox-24, and Ox-72 treatments (Figure S8). Further, CO_2_ emissions were similar throughout the experiment across the Ox-72, Ox-24, and Ox-8 treatments (Figure S9). Consequently, we surmise that the supply of labile organic substrates for Fe reducers did not differ with the different O_2_ pulse lengths in our experiment.

We also tested whether differences in the microbial community composition (particularly Fe reducers) could underpin the differences that we observed in Fe^II^ production rates. The metabolism of Fe reducers is generally thought to be inhibited during oxic conditions due to both competition for reductants with aerobic organisms, which use O_2_ as an electron acceptor, a far more thermodynamically favorable reaction,^40^ and because O_2_ is toxic to many anaerobic organisms and can trigger anaerobes to generate protective enzymes or form cysts.^74^ It is possible that very short pulses of O_2_ (i.e., <0.5 h) in an otherwise anoxic environment may not be sufficient for aerobic organisms to outcompete Fe reducers for reduced-C electron donors, whereas very long exposure to O_2_ (i.e., 2 weeks) might cause more sweeping changes in the microbial community composition and growth efficiency.

**Figure 5:**
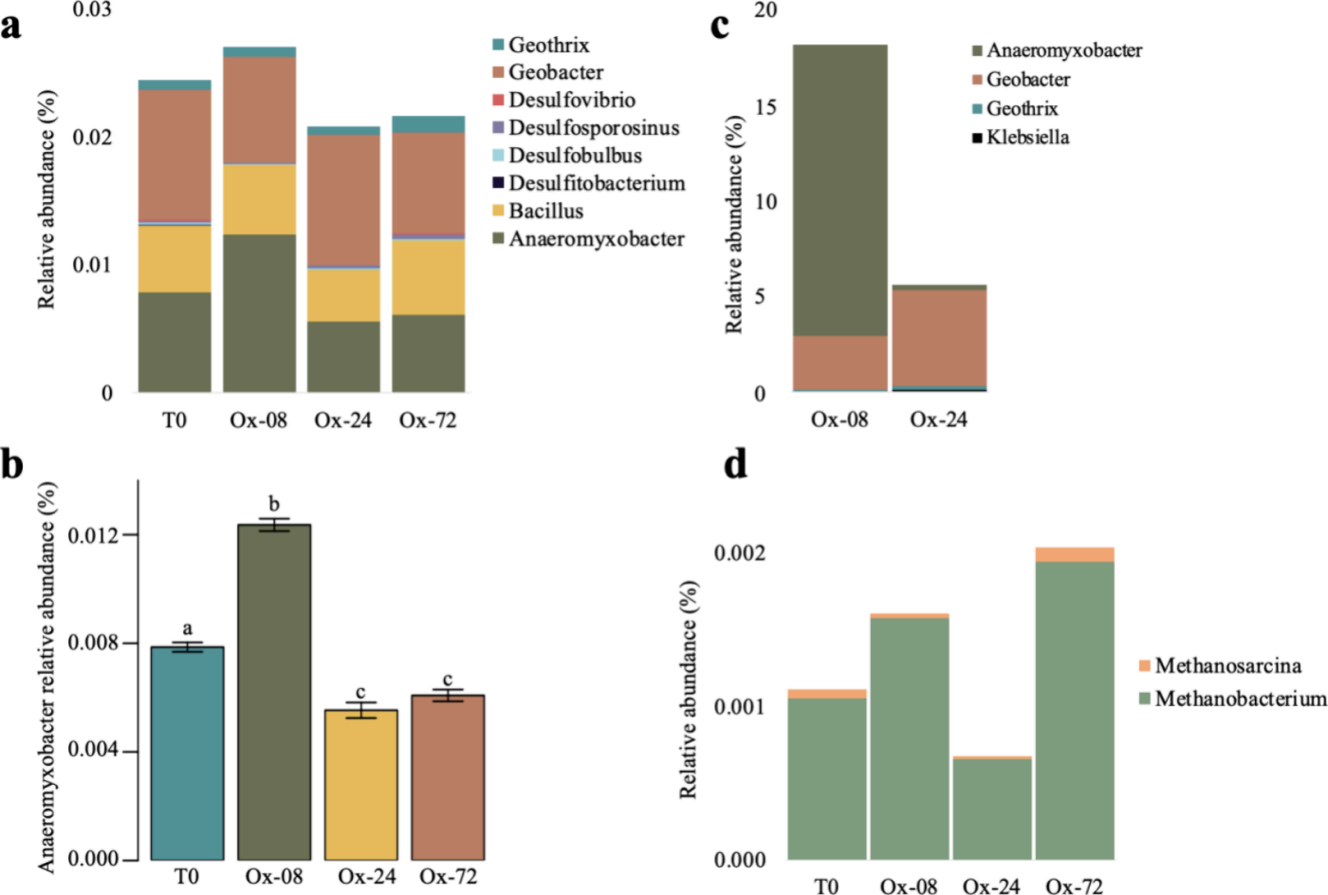
Stacked bar plots of relative sequence abundance of iron reducers and methanogens in a tropical forest soil incubated with redox oscillation treatments. (a) Relative abundance of iron-reducing clades at the beginning of the experiment (*T*_0_, *n* = 3) and 5–6 weeks later, at the end of the last anoxic interval for incubations with a 6-day anoxic interval followed by either 72 h of oxygen exposure (Ox-72; *n* = 2), 24 h of O_2_ (Ox-24; *n* = 2), or 8 h of O_2_ (Ox-8; *n* = 2). (b) Relative abundance of the Anaeromyxobacter bacterial clade. Letter designations indicate the significance of Tukey’s pairwise comparisons. (c) Relative abundance of iron-reducing bacteria that were isotopically enriched after ^13^C/^15^N-labeled amino acids were added in the penultimate cycle of redox oscillation. (d) Relative abundance of methanogens at the beginning of the experiment and the end of the last anoxic interval. All relative sequence abundances are based on 16S rRNA gene amplicons.

In our study, we identified several dominant iron-reducing genera, including Anaeromyxobacter, Geobacter, and Geothrix across all treatments (Figure 5a). The relative abundance of Anaeromyxobacter was significantly higher in the Ox-08 treatment compared to Ox-24 and Ox-72 (*p* < 0.05) (Figure 5b). Additionally, when we used SIP-DNA analysis to assess bacterial activity immediately following exposure to O_2_, Anaeromyxobacter was the most responsive group to ^13^C-amino acid additions, with a relative abundance of 15% in Ox-08, compared to just 0.3% in Ox-24 (Figure 5c). This strongly suggests that Anaeromyxobacter can maintain higher activity following only 8 h of O_2_ exposure relative to 24 h.

Anaeromyxobacteria have been previously detected in LEF soils exposed to slow and fast oxidation rates^34^ and are frequently observed in poorly drained/depressional soils.^75^ Another common Fe reducer that we observed, Geobacter, can tolerate O_2_ exposure over 24 h in pure culture conditions,^76^ but their *in situ* temporal threshold for O_2_ tolerance in soils is unknown. If the threshold for Fe reducers to maintain high activity is between 8 and 24 h for our system, then this could explain the higher Fe reduction rates that we observed in the treatments with shorter τ_oxic_. The ability of Fe reducers to rapidly resume activity after O_2_ exposure or shifts in the availability of reducible Fe^III^ phases could explain the increased anaerobic Fe reduction rates that we observed with decreasing O_2_ exposure time.

Adding ^13^C-amino acids dramatically increased Fe reduction rates (by 9× and 4× for the Ox-24 and Ox-8 treatments, respectively (Figure S10)) and appeared to shift the system from one responding to the length of oxic exposure to one dominated by an external labile carbon pulse. The DNA-SIP data show that the Ox-8 Fe reducers, which were mostly *Anaeromyxobacter*, took up and incorporated more labeled C into their DNA than the Ox-24 Fe reducers, which were mostly *Geobacter* (Figure 5c). The Ox-8 Fe reducers were more active at the time of the C addition; however, the Ox-24 Fe reducers led to higher Fe reduction rates with this new C source (Figure S10). This nuance may reflect a transitory adjustment to the new regime, or perhaps *Geobacter* has a lower growth yield than *Anaeromyxobacter* on the amino acid substrate.

### Longer O_2_ Perturbations Lead to Higher CH_4_ Emissions

In contrast to our Fe reduction results, anaerobic CH_4_ emissions increased when we lengthened the O_2_ exposure intervals (Figures 2 and 3 and Figure S11). While the general pattern of instantaneous CH_4_ flux was similar across treatments (i.e., increasing CH_4_ flux over the 6-day anoxic period followed by a sharp decrease during oxic periods), CH_4_ fluxes were more pronounced in the Ox-72 and Ox-24 treatments than in the Ox-8 treatments (Figure 2b). Cumulative CH_4_ fluxes decreased significantly with decreasing oxic exposure length (Ox-72 > Ox-24 > Ox-8), with the Ox-24 treatment maintaining similar CH_4_ fluxes to the preconditioning cycles (which had 24 h oxic periods) (Figure 3b and Figure S11). Thus, lengthening the oxic exposure to 72 h increased CH_4_ fluxes, while decreasing oxic exposure to 8 h decreased CH_4_ fluxes.

Fe^III^ reduction is well-known to suppress CH_4_ production in soils, as Fe reducers can often outcompete methanogens for key substrates like acetate or H_2_.^37,77^ This is likely why within each anoxic interval, we do not see that CH_4_ emissions begin to increase until Fe reduction rates start to decline (Figures 2 and 3). Higher Fe reduction rates in the shorter τ_oxic_ treatments could thus be expected to suppress CH_4_ emissions more than in longer τ_oxic_ treatments (Figures 2 and 3). To examine this, we plotted rates of Fe^II^ and CH_4_ production for each treatment and found that the Fe^II^:CH_4_ regression slope shifts from 0.29 to 1.32 for the Ox-72 to Ox-8 treatments (Figure S12 and Table S7), consistent with greater anaerobic Fe^II^ production and lower anaerobic CH_4_ fluxes following shorter O_2_ exposure (Figure S13 and Table S7). Fe^II^:CH_4_ regression slopes were statistically significant for preconditioning (0.85), Ox-24 (0.88), and Ox-72 (0.62) but not statistically significant for Ox-8 (1.32) (Figure S12). The generation of rapidly reducible SRO Fe^III^ phases during each oxidation event fuels Fe reducer activity, and our SIP-DNA measurements show that at least the Fe reducer Anaeromyxobacter is more active at the beginning of the anoxic cycle in the 8 h treatment than in the 24 h treatment (the 72 h treatment could not be measured due to the sample loss). As others have separately shown, experimental additions of SRO Fe^III^ to similar Luquillo Experimental Forest (LEF) soils can stimulate iron reducers to outcompete methanogens.^37^ What is less clear is why longer oxic exposure length appears to diminish Fe^II^ production more than CH_4_ emissions.

### Anaerobe Tolerance to O_2_

The ability of anaerobic organisms to tolerate periodic exposure to O_2_ is essential for them to survive and thrive in redox fluctuating environments, and we have shown previously that organisms populating the LEF soils are adapted to frequent redox shifts.^25,34,49^ In our current experiment, it appears that longer O_2_ exposure has a negative effect on the activity of Fe reducers, but that methanogens are not similarly constrained. In fact, in the Ox-72 treatment, where Fe reduction rates were the lowest, CH_4_ fluxes begin to increase immediately upon the initiation of the τ_anoxic_ interval, whereas in the Ox-24 and Ox-8 treatment, CH_4_ fluxes were typically delayed for ∼48 h (Figure 2). We observed *Methanobacterium* as the dominant methanogen in all treatments (Figure 5d); prior work has shown that this taxa can recover rapidly following O_2_ exposure.^78,79^ However, the relative abundance of *Methanobacterium* was exceedingly low (∼0.001%), which may have influenced the lack of significant differences in relative abundance between treatments, as well as our inability to detect methanogen activity at the beginning of the anoxic cycle using SIP-DNA measurements (Figure 5d). Although methanogens are strict anaerobes, recent findings suggest that they can be less constrained by O_2_ exposure than previously assumed,^36,78−80^ with some species producing CH_4_ even when cultured with low levels of O_2_ (up to 1%).^81^ While it is understood that longer O_2_ exposure can lower subsequent anaerobic activity for methanogen cultures,^82^ recovery times can be as short as 1 day^78^ and full viability can often be preserved after week- to month-long exposures to O_2_,^83^ especially when low redox microsites or other anaerobic microbes are present.^37,78,84^ More complex interactions between Fe and CH_4_ might also explain the suppression of CH_4_ fluxes during periods of high Fe reduction, such as the coupling of anaerobic oxidation of CH_4_ to Fe reduction by methanotrophic archaea and bacteria,^85,86^ direct interspecies electron transfer (DIET) processes,^87,88^ or specific substrate preferences and availability, which link Fe reducers, methanotrophs, and methanogens.^89^

### Environmental Implications

Our findings illustrate that the duration of O_2_ exposure can be an important determinant of Fe reduction rates, a fundamental ecosystem process in upland soils. Short periods of O_2_ exposure appear to drive rapid Fe reduction, whereas longer O_2_ exposure can hinder Fe reduction. We found that the duration of O_2_ exposure can affect the balance of Fe reduction and CH_4_ emissions in soils experiencing redox oscillations. For soils exposed to 6 days of anoxia, as O_2_ exposure decreased from 72 to 24 to 8 h, the subsequent anoxic intervals had higher Fe reduction rates and lower CH_4_ emissions, with no change in CO_2_ fluxes.

The ecosystem consequences of variable redox conditions manifest through the timescales and rates of the governing processes.^90^ For instance, a key consequence of a shift from oxic to anoxic conditions is the solubilization of phosphorus,^91−93^ organic matter,^24,94,95^ and various contaminant metals^96−98^ associated with the reductive dissolution of high-surface-area Fe oxides that often sorb these constituents.^99^ However, the release of these constituents is governed by the kinetics of Fe reduction, which can either be sluggish or extremely rapid depending on environmental conditions. Frequent redox fluctuations have been shown previously^19^ and theoretically^28^ to favor high Fe reduction rates, and here, we now show that specifically the length of O_2_ exposure modulates Fe reduction rates. Soil ecosystems that favor short periods of oxygenation of soil microsites should also favor faster Fe reduction rates and greater releases of sorbed constituents.

The implications of an Fe redox cycle that is modulated by O_2_ exposure length could be profound. The principal intersection of the Fe redox cycle with ecosystem function is via its coupling with the C cycle,^7,41,100,101^ although Fe is also a critical elemental sorbent for the key plant nutrient phosphorus.^57,92,102,103^ Incorporating the dynamics of Fe cycles into global ecosystem models has been challenging because it has not been clear how to tie changes in soil moisture to Fe reduction rates.^8,104,105^ In an encouraging step, Calabrese et al.^28^ have shown that the theoretical maximum in cumulative ecosystem Fe reduction will occur when redox fluctuations are as frequent as possible given the growth and activity constraints of microbial Fe reducers. Our results further this theory and we postulate that microbial Fe reducers thrive in environments with relatively short pulses of O_2_, which should be predictable based on rainfall patterns.^22,104,106^

Some studies estimate as much as 50% of the C mineralization in humid soils could be coupled to Fe reduction,^19^ an estimate supported by the theoretical work of Calabrese et al.^28^ Another work^7^ suggests that an acceleration of the Fe reduction/oxidation cycles would likely lead to a net decrease in organic matter persistence by destabilizing Fe-associated organic matter, both directly via Fe reduction and indirectly via Fenton chemistry if oxidation events occur when Fe^II^ concentrations are high. Further, P behavior can become dominated by Fe cycle dynamics in redox dynamic systems^102^ as the oxidation of Fe^II^ generates SRO Fe^III^ phases that sorb phosphorus, which are then subsequently dissolved during reduction events.^92,107−109^

Our study probed the biogeochemical dynamics of a tropical forest soil in the absence of spatial heterogeneity by forcing microbial competition in a slurried soil reactor. Stimulating Fe reduction has long been shown to curtail CH_4_ production in wetlands and soil systems. With spatial heterogeneity minimized, our results suggest methanogens are less affected by longer O_2_ exposure than Fe reducers and thus might have a competitive advantage in systems that become oxygenated for long periods of time, such as through extensive soil drainage or droughts. Conversely, we might expect frequent, short aeration events to minimize CH_4_ production in systems with appreciable Fe redox cycling.

## Supporting Information Available

Four sections comprising 13 additional figures, seven additional tables, and three additional method descriptions (PDF)

## References

[ref1] Lin Y.; Campbell A. N.; Bhattacharyya A.; DiDonato N.; Thompson A. M.; Tfaily M. M.; Nico P. S.; Silver W. L.; Pett-Ridge J. Differential effects of redox conditions on the decomposition of litter and soil organic matter. Biogeochemistry 2021, 154 (1), 1–15. 10.1007/s10533-021-00790-y.

[ref2] Wood T. E.; Detto M.; Silver W. L. Sensitivity of Soil Respiration to Variability in Soil Moisture and Temperature in a Humid Tropical Forest. PLoS One 2013, 8 (12), e80965 10.1371/journal.pone.0080965. 24312508 PMC3846571

[ref3] Wilmoth J. L. Redox Heterogeneity Entangles Soil and Climate Interactions. In Sustainability 2021, 13 (18), 10084 10.3390/su131810084.

[ref4] Zakem E. J.; Polz M. F.; Follows M. J. Redox-informed models of global biogeochemical cycles. Nat. Commun. 2020, 11 (1), 5680 10.1038/s41467-020-19454-w. 33173062 PMC7656242

[ref5] Peiffer S.; Kappler A.; Haderlein S. B.; Schmidt C.; Byrne J. M.; Kleindienst S.; Vogt C.; Richnow H. H.; Obst M.; Angenent L. T.; Bryce C.; McCammon C.; Planer-Friedrich A biogeochemical–hydrological framework for the role of redox-active compounds in aquatic systems. Nature Geoscience 2021, 14 (5), 264–272. 10.1038/s41561-021-00742-z.

[ref6] Levar C. E.; Hoffman C. L.; Dunshee A. J.; Toner B. M.; Bond D. R. Redox potential as a master variable controlling pathways of metal reduction by geobacter sulfurreducens. ISME J. 2017, 11 (3), 741–752. 10.1038/ismej.2016.146. 28045456 PMC5322298

[ref7] Chen C.; Hall S. J.; Coward E.; Thompson A. Iron-mediated organic matter decomposition in humid soils can counteract protection. Nat. Commun. 2020, 11 (1), 2255 10.1038/s41467-020-16071-5. 32382079 PMC7206102

[ref8] Keiluweit M.; Gee K.; Denney A.; Fendorf S. Anoxic microsites in upland soils dominantly controlled by clay content. Soil Biol. Biochem 2018, 118, 42–50. 10.1016/j.soilbio.2017.12.002.

[ref9] Wanzek T.; Keiluweit M.; Baham J.; Dragila M. I.; Fendorf S.; Fiedler S.; Nico P. S.; Kleber M. Quantifying biogeochemical heterogeneity in soil systems. Geoderma 2018, 324, 89–97. 10.1016/j.geoderma.2018.03.003.

[ref10] Sexstone A. J.; Revsbech N. P.; Parkin T. B.; Tiedje J. M. Direct measurement of oxygen profiles and denitrification rates in soil aggregates. Soil Sci. Soc. Am. J. 1985, 49 (3), 645–651. 10.2136/sssaj1985.03615995004900030024x.

[ref11] Keiluweit M.; Nico P. S.; Kleber M.; Fendorf S. Are oxygen limitations under recognized regulators of organic carbon turnover in upland soils?. Biogeochemistry 2016, 127 (2–3), 157–171. 10.1007/s10533-015-0180-6.

[ref12] Lacroix E. M.; Rossi R. J.; Bossio D.; Fendorf S. Effects of moisture and physical disturbance on pore-scale oxygen content and anaerobic metabolisms in upland soils. Science of The Total Environment 2021, 780, 146572 10.1016/j.scitotenv.2021.146572. 33774307

[ref13] Lacroix E. M.; Masue-Slowey Y.; Dlott G. A.; Keiluweit M.; Chadwick O. A.; Fendorf S. Mineral Protection and Resource Limitations Combine to Explain Profile-Scale Soil Carbon Persistence. J. Geophys. Res.: Biogeosci. 2022, 127 (4), e2021JG006674 10.1029/2021JG006674.

[ref14] Franklin S.; Vasilas B.; Jin Y. More than Meets the Dye: Evaluating Preferential Flow Paths as Microbial Hotspots. Vadose Zone J. 2019, 18 (1), 190024 10.2136/vzj2019.03.0024.

[ref15] Chen C.; Barcellos D.; Richter D. D.; Schroeder P. A.; Thompson A. Redoximorphic Bt horizons of the Calhoun CZO soils exhibit depth-dependent iron-oxide crystallinity. Journal of Soils and Sediments 2019, 19, 785–797. 10.1007/s11368-018-2068-2.

[ref16] Growle A. J.; Lueker D. C.; Gaskill H. S. Periodic (Liesegang) Precipitation of Chemicals. Nature 1963, 199 (4893), 623–624. 10.1038/199623b0. 14072028

[ref17] Gasparatos D.; Tarenidis D.; Haidouti C.; Oikonomou G. Microscopic structure of soil Fe-Mn nodules: environmental implication. Environmental Chemistry Letters 2005, 2 (4), 175–178. 10.1007/s10311-004-0092-5.

[ref18] Dong X.; Richter D. D.; Thompson A.; Wang J. The primacy of temporal dynamics in driving spatial self-organization of soil iron redox patterns. Proc. Natl. Acad. Sci. U. S. A. 2023, 120 (51), e2313487120 10.1073/pnas.2313487120. 38096416 PMC10742380

[ref19] Barcellos D.; Cyle K. T.; Thompson A. Faster redox fluctuations can lead to higher iron reduction rates in humid forest soils. Biogeochemistry 2018, 137 (3), 367–378. 10.1007/s10533-018-0427-0.

[ref20] Liptzin D.; Silver W. L.; Detto M. Temporal Dynamics in Soil Oxygen and Greenhouse Gases in Two Humid Tropical Forests. Ecosystems 2011, 14 (2), 171–182. 10.1007/s10021-010-9402-x.

[ref21] Hall S. J.; McDowell W. H.; Silver W. L. When wet gets wetter: decoupling of moisture, redox biogeochemistry, and greenhouse gas fluxes in a humid tropical forest soil. Ecosystems 2013, 16 (4), 576–589. 10.1007/s10021-012-9631-2.

[ref22] Barcellos D.; O’Connell C.; Silver W.; Meile C.; Thompson A. Hot Spots and Hot Moments of Soil Moisture Explain Fluctuations in Iron and Carbon Cycling in a Humid Tropical Forest Soil. Soil Systems 2018, 2 (4), 59 10.3390/soilsystems2040059.

[ref23] DeAngelis K. M.; Silver W. L.; Thompson A. W.; Firestone M. K. Microbial communities acclimate to recurring changes in soil redox potential status. Environmental Microbiology 2010, 12 (12), 3137–3149. 10.1111/j.1462-2920.2010.02286.x. 20629704

[ref24] Bhattacharyya A.; Campbell A. N.; Tfaily M. M.; Lin Y.; Kukkadapu R. K.; Silver W. L.; Nico P. S.; Pett-Ridge J. Redox Fluctuations Control the Coupled Cycling of Iron and Carbon in Tropical Forest Soils. Environ. Sci. Technol. 2018, 52 (24), 14129–14139. 10.1021/acs.est.8b03408. 30451506

[ref25] Pett-Ridge J.; Silver W. L.; Firestone M. K. Redox fluctuations frame microbial community impacts on N-cycling rates in a humid tropical forest soil. Biogeochemistry 2006, 81 (1), 95–110. 10.1007/s10533-006-9032-8.

[ref26] Pett-Ridge J.; Petersen D. G.; Nuccio E.; Firestone M. K. Influence of oxic/anoxic fluctuations on ammonia oxidizers and nitrification potential in a wet tropical soil. FEMS Microbiology Ecology 2013, 85 (1), 179–194. 10.1111/1574-6941.12111. 23556538

[ref27] Pett-Ridge J. Rapidly fluctuating redox regimes frame the ecology of microbial communities and their biogeochemical function in a humid tropical soil. Doctoral Dissertation, University of California - Berkeley: Berkeley, CA, USA, 2005.

[ref28] Calabrese S.; Barcellos D.; Thompson A.; Porporato A. Theoretical Constraints on Fe Reduction Rates in Upland Soils as a Function of Hydroclimatic Conditions. J. Geophys. Res.: Biogeosci. 2020, 125 (12), e2020JG005894 10.1029/2020JG005894.

[ref29] Zhang Z.; Furman A. Soil redox dynamics under dynamic hydrologic regimes - A review. Science of The Total Environment 2021, 763, 143026 10.1016/j.scitotenv.2020.143026. 33143917

[ref30] Bonaiuti S.; Blodau C.; Knorr K.-H. Transport, anoxia and end-product accumulation control carbon dioxide and methane production and release in peat soils. Biogeochemistry 2017, 133 (2), 219–239. 10.1007/s10533-017-0328-7.

[ref31] Chen C.; Meile C.; Wilmoth J. L.; Barcellos D.; Thompson A. Influence of pO2 on Iron Redox Cycling and Anaerobic Organic Carbon Mineralization in a Humid Tropical Forest Soil. Environ. Sci. Technol. 2018, 52 (14), 7709–7719. 10.1021/acs.est.8b01368. 29890827

[ref32] Lancellotti B. V.; Underwood K. L.; Perdrial J. N.; Schroth A. W.; Roy E. D.; Adair C. E. Complex Drivers of Riparian Soil Oxygen Variability Revealed Using Self-Organizing Maps. Water Resour. Res. 2023, 59 (6), e2022WR034022 10.1029/2022WR034022.

[ref33] Ginn B.; Meile C.; Wilmoth J.; Tang Y.; Thompson A. Rapid Iron Reduction Rates Are Stimulated by High-Amplitude Redox Fluctuations in a Tropical Forest Soil. Environ. Sci. Technol. 2017, 51 (6), 3250–3259. 10.1021/acs.est.6b05709. 28244747

[ref34] Wilmoth J. L.; Moran M. A.; Thompson A. Transient O2 pulses direct Fe crystallinity and Fe(III)-reducer gene expression within a soil microbiome. Microbiome 2018, 6 (1), 189 10.1186/s40168-018-0574-5. 30352628 PMC6199725

[ref35] Gabriel G. V. M.; Oliveira L. C.; Barros D. J.; Bento M. S.; Neu V.; Toppa R. H.; Carmo J. B.; Navarrete A. A. Methane emission suppression in flooded soil from Amazonia. Chemosphere 2020, 250, 126263 10.1016/j.chemosphere.2020.126263. 32088616

[ref36] Angle J. C.; Morin T. H.; Solden L. M.; Narrowe A. B.; Smith G. J.; Borton M. A.; Rey-Sanchez C.; Daly R. A.; Mirfenderesgi G.; Hoyt D. W.; Riley W. J.; Miller C. S.; Bohrer G.; Wrighton K. C. Methanogenesis in oxygenated soils is a substantial fraction of wetland methane emissions. Nat. Commun. 2017, 8 (1), 1567 10.1038/s41467-017-01753-4. 29146959 PMC5691036

[ref37] Teh Y. A.; Dubinsky E. A.; Silver W. L.; Carlson C. M. Suppression of methanogenesis by dissimilatory Fe (III)-reducing bacteria in tropical rain forest soils: Implications for ecosystem methane flux. Global Change Biology 2008, 14 (2), 413–422. 10.1111/j.1365-2486.2007.01487.x.

[ref38] Hu J.; Wu H.; Sun Z.; Peng Q.-a.; Zhao J.; Hu R. Ferrous iron (Fe2+) addition decreases methane emissions induced by rice straw in flooded paddy soils. ACS Earth and Space Chemistry 2020, 4 (6), 843–853. 10.1021/acsearthspacechem.0c00024.

[ref39] Sivan O.; Shusta S.; Valentine D. Methanogens rapidly transition from methane production to iron reduction. Geobiology 2016, 14 (2), 190–203. 10.1111/gbi.12172. 26762691

[ref40] Becking L. G. M. B.; Kaplan I. R.; Moore D. Limits of the Natural Environment in Terms of pH and Oxidation-Reduction Potentials. Journal of Geology 1960, 68 (3), 243–284. 10.1086/626659.

[ref41] Lovley D. R.; Phillips E. J. Novel mode of microbial energy metabolism: organic carbon oxidation coupled to dissimilatory reduction of iron or manganese. Applied and environmental microbiology 1988, 54 (6), 1472–1480. 10.1128/aem.54.6.1472-1480.1988. 16347658 PMC202682

[ref42] LaRowe D. E.; Van Cappellen P. Degradation of natural organic matter: a thermodynamic analysis. Geochim. Cosmochim. Acta 2011, 75 (8), 2030–2042. 10.1016/j.gca.2011.01.020.

[ref43] Roden E. E. Microbial iron-redox cycling in subsurface environments. Biochem. Soc. Trans. 2012, 40 (6), 1249–1256. 10.1042/BST20120202. 23176463

[ref44] Masiello C. A.; Gallagher M. E.; Randerson J. T.; Deco R. M.; Chadwick O. A. Evaluating two experimental approaches for measuring ecosystem carbon oxidation state and oxidative ratio. J. Geophys. Res.: Biogeosci. 2008, 113 (G3), C03010.1 10.1029/2007JG000534.

[ref45] Boye K.; Noël V.; Tfaily M. M.; Bone S. E.; Williams K. H.; Bargar J. R.; Fendorf S. Thermodynamically controlled preservation of organic carbon in floodplains. Nat. Geosci. 2017, 10 (6), 415–419. 10.1038/ngeo2940.

[ref46] Keiluweit M.; Wanzek T.; Kleber M.; Nico P.; Fendorf S. Anaerobic microsites have an unaccounted role in soil carbon stabilization. Nat. Commun. 2017, 8 (1), 1771 10.1038/s41467-017-01406-6. 29176641 PMC5701132

[ref47] Bhattacharyya A.; Kukkadapu R. K.; Bowden M.; Pett-Ridge J.; Nico P. S. Fast redox switches lead to rapid transformation of goethite in humid tropical soils: A Mössbauer spectroscopy study. Soil Science Society of America Journal 2022, 86 (2), 264–274. 10.1002/saj2.20382.

[ref48] Laio F.; Porporato A.; Ridolfi L.; Rodriguez-Iturbe I. Plants in water-controlled ecosystems: active role in hydrologic processes and response to water stress: II. Probabilistic soil moisture dynamics. Advances in Water Resources 2001, 24 (7), 707–723. 10.1016/S0309-1708(01)00005-7.

[ref49] Pett-Ridge J.; Firestone M. Redox fluctuation structures microbial communities in a wet tropical soil. Applied and environmental microbiology 2005, 71 (11), 6998–7007. 10.1128/AEM.71.11.6998-7007.2005. 16269735 PMC1287741

[ref50] Mason-Jones K.; Robinson S. L.; Veen G. F.; Manzoni S.; van der Putten W. H. Microbial storage and its implications for soil ecology. ISME Journal 2022, 16 (3), 617–629. 10.1038/s41396-021-01110-w. 34593996 PMC8857262

[ref51] Conrad R. Soil microorganisms as controllers of atmospheric trace gases (H2, CO, CH4, OCS, N2O, and NO). Microbiological Reviews 1996, 60 (4), 609–640. 10.1128/mr.60.4.609-640.1996. 8987358 PMC239458

[ref52] Roden E. E. Diversion of Electron Flow from Methanogenesis to Crystalline Fe(III) Oxide Reduction in Carbon-Limited Cultures of Wetland Sediment Microorganisms. Appl. Environ. Microbiol. 2003, 69 (9), 5702 10.1128/AEM.69.9.5702-5706.2003. 12957966 PMC194912

[ref53] Bar-Or I.; Elvert M.; Eckert W.; Kushmaro A.; Vigderovich H.; Zhu Q.; Ben-Dov E.; Sivan O. Iron-Coupled Anaerobic Oxidation of Methane Performed by a Mixed Bacterial-Archaeal Community Based on Poorly Reactive Minerals. Environ. Sci. Technol. 2017, 51 (21), 12293–12301. 10.1021/acs.est.7b03126. 28965392

[ref54] Ettwig K. F.; Zhu B.; Speth D.; Keltjens J. T.; Jetten M. S.; Kartal B. Archaea catalyze iron-dependent anaerobic oxidation of methane. Proc. Natl. Acad. Sci. U. S. A. 2016, 113 (45), 12792–12796. 10.1073/pnas.1609534113. 27791118 PMC5111651

[ref55] Blazewicz S. J.; Petersen D. G.; Waldrop M. P.; Firestone M. K. Anaerobic oxidation of methane in tropical and boreal soils: ecological significance in terrestrial methane cycling. J. Geophys. Res.: Biogeosci. 2012, 117 (G2), G02033.1 10.1029/2011JG001864.

[ref56] Ginn B. R.; Habteselassie M. Y.; Meile C.; Thompson A. Effects of sample storage on microbial Fe-reduction in tropical rainforest soils. Soil Biology and Biochemistry 2014, 68, 44–51. 10.1016/j.soilbio.2013.09.012.

[ref57] Peretyazhko T.; Sposito G. Iron (III) reduction and phosphorous solubilization in humid tropical forest soils. Geochim. Cosmochim. Acta 2005, 69 (14), 3643–3652. 10.1016/j.gca.2005.03.045.

[ref58] Tishchenko V.; Meile C.; Scherer M. M.; Pasakarnis T. S.; Thompson A. Fe2+ catalyzed iron atom exchange and re-crystallization in a tropical soil. Geochim. Cosmochim. Acta 2015, 148, 191–202. 10.1016/j.gca.2014.09.018.

[ref59] Barcellos D. Biogeochemical cycling of iron and carbon in humid (sub)tropical forest soils under fluctuating redox conditions. Doctoral Dissertation, University of Georgia: Athens, GA, USA, 2018.

[ref60] Thompson A.; Chadwick O. A.; Rancourt D. G.; Chorover J. Iron-oxide crystallinity increases during soil redox oscillations. Geochim. Cosmochim. Acta 2006, 70 (7), 1710–1727. 10.1016/j.gca.2005.12.005.

[ref61] Parada A. E.; Needham D. M.; Fuhrman J. A. Every base matters: assessing small subunit rRNA primers for marine microbiomes with mock communities, time series and global field samples. Environmental Microbiology 2016, 18 (5), 1403–1414. 10.1111/1462-2920.13023. 26271760

[ref62] Apprill A.; McNally S.; Parsons R.; Weber L. Minor revision to V4 region SSU rRNA 806R gene primer greatly increases detection of SAR11 bacterioplankton. Aquatic Microbial Ecology 2015, 75 (2), 129–137. 10.3354/ame01753.

[ref63] Nuccio E. E.; Blazewicz S. J.; Lafler M.; Campbell A. N.; Kakouridis A.; Kimbrel J. A.; Wollard J.; Vyshenska D.; Riley R.; Tomatsu A.; Hestrin R.; Malmstrom R. R.; Firestone M.; Pett-Ridge J. HT-SIP: a semi-automated stable isotope probing pipeline identifies cross-kingdom interactions in the hyphosphere of arbuscular mycorrhizal fungi. Microbiome 2022, 10 (1), 199 10.1186/s40168-022-01391-z. 36434737 PMC9700909

[ref64] Callahan B. J.; McMurdie P. J.; Rosen M. J.; Han A. W.; Johnson A. J. A.; Holmes S. P. DADA2: High-resolution sample inference from Illumina amplicon data. Nat. Methods 2016, 13 (7), 581–583. 10.1038/nmeth.3869. 27214047 PMC4927377

[ref65] Halekoh U.; Ho̷jsgaard S. A kenward-roger approximation and parametric bootstrap methods for tests in linear mixed models–the R package pbkrtest. J. Stat. Software 2014, 59 (9), 1–32. 10.18637/jss.v059.i09.

[ref66] Bates D.; Mächler M.; Bolker B.; Walker S. Fitting Linear Mixed-Effects Models Using lme4. J. Stat. Software 2015, 67 (1), 48 10.18637/jss.v067.i01.

[ref67] Parks D. H.; Tyson G. W.; Hugenholtz P.; Beiko R. G. STAMP: statistical analysis of taxonomic and functional profiles. Bioinformatics 2014, 30 (21), 3123–3124. 10.1093/bioinformatics/btu494. 25061070 PMC4609014

[ref68] Hall S. J.; Silver W. L. Reducing conditions, reactive metals, and their interactions can explain spatial patterns of surface soil carbon in a humid tropical forest. Biogeochemistry 2015, 125 (2), 149–165. 10.1007/s10533-015-0120-5.

[ref69] Kappler A.; Bryce C.; Mansor M.; Lueder U.; Byrne J. M.; Swanner E. D. An evolving view on biogeochemical cycling of iron. Nature Reviews Microbiology 2021, 19 (6), 360–374. 10.1038/s41579-020-00502-7. 33526911

[ref70] Mikutta C.; Niegisch M.; Thompson A.; Behrens R.; Schnee L. S.; Hoppe M.; Dohrmann R. Redox cycling of straw-amended soil simultaneously increases iron oxide crystallinity and the content of highly disordered organo-iron(III) solids. Geochim. Cosmochim. Acta 2024, 371, 126–143. 10.1016/j.gca.2024.02.009.

[ref71] Chen C.; Thompson A. Ferrous Iron Oxidation under Varying pO2 Levels: The Effect of Fe (III)/Al (III) Oxide Minerals and Organic Matter. Environ. Sci. Technol. 2018, 52 (2), 597–606. 10.1021/acs.est.7b05102. 29192502

[ref72] Schwaminger S. P.; Surya R.; Filser S.; Wimmer A.; Weigl F.; Fraga-García P.; Berensmeier S. Formation of iron oxide nanoparticles for the photooxidation of water: Alteration of finite size effects from ferrihydrite to hematite. Sci. Rep. 2017, 7 (1), 12609 10.1038/s41598-017-12791-9. 28974753 PMC5626691

[ref73] Hu Y.; Li Q.; Lee B.; Jun Y.-S. Aluminum affects heterogeneous Fe (III)(Hydr) oxide nucleation, growth, and ostwald ripening. Environ. Sci. Technol. 2014, 48 (1), 299–306. 10.1021/es403777w. 24289329

[ref74] Gambrell R. P.; DeLaune R. D.; Patrick W. H. Jr. Redox processes in soils following oxygen depletion. In Plant Life Under Oxygen Deprivation; SPB Academic Publishing BV: The Hague, The Netherlands, 1991, 101–117.

[ref75] Suriyavirun N.; Krichels A. H.; Kent A. D.; Yang W. H. Microtopographic differences in soil properties and microbial community composition at the field scale. Soil Biology and Biochemistry 2019, 131, 71–80. 10.1016/j.soilbio.2018.12.024.

[ref76] Lin W. C.; Coppi M. V.; Lovley D. R. Geobacter sulfurreducens Can Grow with Oxygen as a Terminal Electron Acceptor. Appl. Environ. Microbiol. 2004, 70 (4), 2525–2528. 10.1128/AEM.70.4.2525-2528.2004. 15066854 PMC383164

[ref77] Roden E. E.; Wetzel R. G. Organic carbon oxidation and suppression of methane production by microbial Fe (III) oxide reduction in vegetated and unvegetated freshwater wetland sediments. Limnology and Oceanography 1996, 41 (8), 1733–1748. 10.4319/lo.1996.41.8.1733.

[ref78] Liu C.-T.; Miyaki T.; Aono T.; Oyaizu H. Evaluation of methanogenic strains and their ability to endure aeration and water stress. Current microbiology 2008, 56 (3), 214–218. 10.1007/s00284-007-9059-7. 17990030

[ref79] Horne A. J.; Lessner D. J. Assessment of the oxidant tolerance of Methanosarcina acetivorans. FEMS Microbiology Letters 2013, 343 (1), 13–19. 10.1111/1574-6968.12115. 23448147 PMC3651780

[ref80] Watanabe T.; Asakawa S.; Hayano K. Long-term submergence of non-methanogenic oxic upland field soils helps to develop the methanogenic archaeal community as revealed by pot and field experiments. Pedosphere 2020, 30 (1), 62–72. 10.1016/S1002-0160(19)60819-2.

[ref81] Jasso-Chávez R.; Santiago-Martínez M. G.; Lira-Silva E.; Pineda E.; Zepeda-Rodríguez A.; Belmont-Díaz J.; Encalada R.; Saavedra E.; Moreno-Sánchez R. Air-Adapted Methanosarcina acetivorans Shows High Methane Production and Develops Resistance against Oxygen Stress. PLoS One 2015, 10 (2), e0117331 10.1371/journal.pone.0117331. 25706146 PMC4338226

[ref82] Wu X. L.; Conrad R. Functional and structural response of a cellulose-degrading methanogenic microbial community to multiple aeration stress at two different temperatures. Environmental Microbiology 2001, 3 (6), 355–362. 10.1046/j.1462-2920.2001.00199.x. 11472500

[ref83] Fetzer S.; Bak F.; Conrad R. Sensitivity of methanogenic bacteria from paddy soil to oxygen and desiccation. FEMS Microbiol. Ecol. 2006, 12 (2), 107–115. 10.1111/j.1574-6941.1993.tb00022.x.

[ref84] Wagner D.; Pfeiffer E. M.; Bock E. Methane production in aerated marshland and model soils: effects of microflora and soil texture. Soil Biology and Biochemistry 1999, 31 (7), 999–1006. 10.1016/S0038-0717(99)00011-5.

[ref85] Valenzuela E. I.; Prieto-Davó A.; López-Lozano N. E.; Hernández-Eligio A.; Vega-Alvarado L.; Juárez K.; García-González A. S.; López M. G.; Cervantes F. J. Anaerobic methane oxidation driven by microbial reduction of natural organic matter in a tropical wetland. Appl. Environ. Microbiol. 2017, 83 (11), e0064517 10.1128/AEM.00645-17. PMC5440706 28341676

[ref86] Aromokeye D. A.; Kulkarni A. C.; Elvert M.; Wegener G.; Henkel S.; Coffinet S.; Eickhorst T.; Oni O. E.; Richter-Heitmann T.; Schnakenberg A.; Taubner H.; Wunder L.; Yin X.; Zhu Q.; Hinrichs K.; Kasten S.; Friedrich M. W. Rates and Microbial Players of Iron-Driven Anaerobic Oxidation of Methane in Methanic Marine Sediments. Front. Microbiol. 2020, 10, 3041 10.3389/fmicb.2019.03041. 32010098 PMC6979488

[ref87] Lovley D. R. Happy together: microbial communities that hook up to swap electrons. ISME journal 2017, 11 (2), 327 10.1038/ismej.2016.136. 27801905 PMC5270577

[ref88] Wegener G.; Krukenberg V.; Riedel D.; Tegetmeyer H. E.; Boetius A. Intercellular wiring enables electron transfer between methanotrophic archaea and bacteria. Nature 2015, 526 (7574), 587 10.1038/nature15733. 26490622

[ref89] He Q.; Yu L.; Li J.; He D.; Cai X.; Zhou S. Electron shuttles enhance anaerobic oxidation of methane coupled to iron(III) reduction. Science of The Total Environment 2019, 688, 664–672. 10.1016/j.scitotenv.2019.06.299. 31254832

[ref90] Rose A. L.; Waite T. D. Predicting iron speciation in coastal waters from the kinetics of sunlight-mediated iron redox cycling. Aquatic Sciences 2003, 65 (4), 375–383. 10.1007/s00027-003-0676-3.

[ref91] Lin Y.; Gross A.; Silver W. L. Low Redox Decreases Potential Phosphorus Limitation on Soil Biogeochemical Cycling Along a Tropical Rainfall Gradient. Ecosystems 2022, 25 (2), 387–403. 10.1007/s10021-021-00662-4.

[ref92] Queiroz H. M.; Ferreira T. O.; Barcellos D.; Nóbrega G. N.; Antelo J.; Otero X. L.; Bernardino A. F. From sinks to sources: The role of Fe oxyhydroxide transformations on phosphorus dynamics in estuarine soils. Journal of Environmental Management 2021, 278, 111575 10.1016/j.jenvman.2020.111575. 33147526

[ref93] Nóbrega G. N.; Otero X. L.; Macías F.; Ferreira T. O. Phosphorus geochemistry in a Brazilian semiarid mangrove soil affected by shrimp farm effluents. Environmental Monitoring and Assessment 2014, 186 (9), 5749–5762. 10.1007/s10661-014-3817-3. 24838803

[ref94] Huang W.; Hall S. J. Elevated moisture stimulates carbon loss from mineral soils by releasing protected organic matter. Nat. Commun. 2017, 8 (1), 1774 10.1038/s41467-017-01998-z. 29176688 PMC5701196

[ref95] LaCroix R. E.; Tfaily M. M.; McCreight M.; Jones M. E.; Spokas L.; Keiluweit M. Shifting mineral and redox controls on carbon cycling in seasonally flooded mineral soils. Biogeosciences 2019, 16 (13), 2573–2589. 10.5194/bg-16-2573-2019.

[ref96] Couture R.-M.; Charlet L.; Markelova E.; Madé B. t.; Parsons C. T. On–off mobilization of contaminants in soils during redox oscillations. Environ. Sci. Technol. 2015, 49 (5), 3015–3023. 10.1021/es5061879. 25633742

[ref97] Barcellos D.; Queiroz H. M.; Ferreira A. D.; Bernardino A. F.; Nóbrega G. N.; Otero X. L.; Ferreira T. O. Short-term Fe reduction and metal dynamics in estuarine soils impacted by Fe-rich mine tailings. Appl. Geochem. 2022, 136, 105134–105134. 10.1016/j.apgeochem.2021.105134.

[ref98] Ferreira A. D.; Duckworth O. W.; Queiroz H. M.; Nóbrega G. N.; Barcellos D.; Bernardino Â. F.; Otero X. L.; Ferreira T. O. Seasonal drives on potentially toxic elements dynamics in a tropical estuary impacted by mine tailings. Journal of Hazardous Materials 2024, 474, 134592 10.1016/j.jhazmat.2024.134592. 38805820

[ref99] Borch T.; Kretzschmar R.; Kappler A.; Cappellen P. V.; Ginder-Vogel M.; Voegelin A.; Campbell K. Biogeochemical redox processes and their impact on contaminant dynamics. Environ. Sci. Technol. 2010, 44 (1), 15–23. 10.1021/es9026248. 20000681

[ref100] Hall S. J.; Liptzin D.; Buss H. L.; DeAngelis K.; Silver W. L. Drivers and patterns of iron redox cycling from surface to bedrock in a deep tropical forest soil: a new conceptual model. Biogeochemistry 2016, 130 (1–2), 177–190. 10.1007/s10533-016-0251-3.

[ref101] Hall S. J.; Silver W. L. Iron oxidation stimulates organic matter decomposition in humid tropical forest soils. Global change biology 2013, 19 (9), 2804–2813. 10.1111/gcb.12229. 23606589

[ref102] Lin Y.; Bhattacharyya A.; Campbell A. N.; Nico P. S.; Pett-Ridge J.; Silver W. L. Phosphorus Fractionation Responds to Dynamic Redox Conditions in a Humid Tropical Forest Soil. Journal of Geophysical Research: Biogeosciences 2018, 123 (9), 3016–3027. 10.1029/2018JG004420.

[ref103] Basinski J. J.; Bone S. E.; Klein A. R.; Thongsomboon W.; Mitchell V.; Shukle J. T.; Druschel G. K.; Thompson A.; Aristilde L. Unraveling iron oxides as abiotic catalysts of organic phosphorus recycling in soil and sediment matrices. Nat. Commun. 2024, 15 (1), 5930 10.1038/s41467-024-47931-z. 39025840 PMC11258345

[ref104] Silver W. L.; Lugo A. E.; Keller M. Soil oxygen availability and biogeochemistry along rainfall and topographic gradients in upland wet tropical forest soils. Biogeochemistry 1999, 44 (3), 301–328. 10.1007/BF00996995.

[ref105] O’Connell C. S.; Ruan L.; Silver W. L. Drought drives rapid shifts in tropical rainforest soil biogeochemistry and greenhouse gas emissions. Nat. Commun. 2018, 9 (1), 1348 10.1038/s41467-018-03352-3. 29632326 PMC5890268

[ref106] Dubinsky E. A.; Silver W. L.; Firestone M. K. Tropical forest soil microbial communities couple iron and carbon biogeochemistry. Ecology 2010, 91 (9), 2604–2612. 10.1890/09-1365.1. 20957955

[ref107] Hall S. J.; Berhe A. A.; Thompson A. Order from disorder: do soil organic matter composition and turnover co-vary with iron phase crystallinity?. Biogeochemistry 2018, 140 (1), 93–110. 10.1007/s10533-018-0476-4.

[ref108] Lin Y.; Gross A.; O’Connell C. S.; Silver W. L. Anoxic conditions maintained high phosphorus sorption in humid tropical forest soils. Biogeosciences 2020, 17 (1), 89–101. 10.5194/bg-17-89-2020.

[ref109] Shaheen S. M.; Wang J.; Baumann K.; Ahmed A. A.; Hsu L.-C.; Liu Y.-T.; Wang S.-L.; Kühn O.; Leinweber P.; Rinklebe J. Stepwise redox changes alter the speciation and mobilization of phosphorus in hydromorphic soils. Chemosphere 2022, 288, 132652 10.1016/j.chemosphere.2021.132652. 34695481

