# Supplementary material for: Duration of O2 Exposure Determines Dominance of FeII vs CH4 Production in Tropical Forest Soils
Source: Environ Sci Technol. 2025 Feb 28;59(9):4469–81. doi: 10.1021/acs.est.4c12329 (PMC11912342; doi:10.1021/acs.est.4c12329)
Supplement: Supplementary file 1 — es4c12329_si_001.pdf [file es4c12329_si_001.pdf]

Supplementary material

Duration of O_2 exposure determines dominance of Fe^{II} vs CH_4 production in tropical forest soils

*Diego Barcellos [a,b], Sherlynette Pérez Castro [a], Ashley Campbell [c,d], Jeffrey A Kimbrel [c], Steven Joseph Blazewicz [c], Jessica Wollard [c], Jennifer Pett-Ridge [c,e,f], and Aaron Thompson [a]**

[a] Department of Crop and Soil Sciences, University of Georgia. Athens, GA 30605, USA

[b] Department of Environmental Sciences, Federal University of São Paulo (UNIFESP), Diadema, São Paulo 09913, Brazil.

[c] Physical and Life Sciences Directorate, Lawrence Livermore National Laboratory. Livermore, CA 94550, USA

[d] Adaptive Biotechnologies, Seattle, 98109, WA, USA

[e] Life & Environmental Sciences Department, University of California, Merced, CA, 95343, USA

[f] Innovative Genomics Institute, University of California, Berkeley, CA, 94720, USA

---

*Corresponding author: Aaron Thompson, University of Georgia, Department of Crop and Soil Sciences, Athens, GA 30602, Email: AaronT@uga.edu

Summary of Supporting Information Contents:

Number of pages: 28, Number of additional methods sections: 3, Number of tables: 7, Number of figures: 13

SECTION 1

MÖSSBAUER SPECTROSCOPY FOR THE SOILS UNDER REDOX OSCILLATIONS

The details regarding the analysis for Mössbauer Spectroscopy was adapted from Chen et al. [1], at the temperatures 50 K, 35 K, 25 K, 13 K, and 5 K. For the soil samples collected at the end of the last (third) oxic interval of the treatments Ox-72, Ox-24, Ox-8, and for the soil at the beginning of the experiment (initial soil), we performed Mössbauer spectral fitting by using the Voigt-based fitting method of Rancourt and Ping (1991) as implemented in the Recoil™ software. For each Fe mineral phase, the relative abundance was obtained from the spectral fitting as a fraction of the total Fe spectral area. All errors for Mossbauer fitting parameters were acquired as two-standard deviation (2σ) errors, computed by Recoil™. This presumes equal Mossbauer recoilless fractions to compute the abundance of all Fe phases detected.

The Mössbauer spectra represents a Fe-bearing solid phase or correlates to a cluster of unresolved solid phase-Fe. The spectral components may form a doublet, sextet, octet, or a collapsed sextet, which indicates a solid phase near the temperature for magnetic ordering temperature (T_N). Solid phase-Fe display an intermediate shape between a doublet and full sextet at the temperature near T_N, filling the area between the superior baseline and the inverse depressions of the peaks. Our approach was to use a separate collapsed sextet component (containing exceedingly large line widths and Bhf = 0 T).

Across the five collected temperatures (50 K, 35 K, 25 K, 13 K, and 5 K), we determined six distinct spectral components. The resolved spectral components and assignments are the following: (1) **Q-Fe^{III}-1,** the deep central doublet, corresponding to Fe^{III} in aluminosilicates or organic matter; (2) **QFe^{II}-1**, the wide ferrous doublet corresponding to adsorbed Fe^{II} or Fe^{II} in clays/organic matter (green line); (3) **Q-Fe^{II}-2** the narrow ferrous doublet corresponding to Fe^{II} in ilmenite (brown line); (4) **HFD-OxHy-1**, the dominant sextet, corresponding to Fe^{III}-oxyhydroxides that are magnetically ordered; (5) **HFD-(b)OxHy** the collapsed 'sextet',

corresponding to Fe^{III} oxyhyroxides near their blocking temperature; and (6) H-(b)Fe^{II} partially magnetically ordered Fe^{II} phase. Mössbauer spectra for each sample are presented in Figures S3 to S6, and detailed fitting parameters are provided in Tables S2 to S5.

SECTION 2
METHODS FOR MICROBIAL ANALYSIS

DNA extraction

DNA was extracted at LLNL using lysing matrix E tubes by combining 0.25 g soil slurry sample with 0.5 mL extraction buffer (350mM K-PO_4, 0.7M NaCl, 50mM EDTA), 0.5 mL equilibrated phenol:chloroform:isoamyl alcohol (25:24:1, pH8), 30uL β-Me, and 20uL bovine serum albumin (BSA) (400mg/mL). Cells were lysed via bead beating on a FastPrep-24 high-speed homogenizer (MP Biomedicals) for 45 sec at 6.5 m s^{-1}. Following lysis, 0.7 M NaCl and CTAB/NaCl (1:10 volume) were added, mixed, and the mixture was cooled on ice for 1 min before centrifugation at 16,000 x *g* for 5 minutes at 4°C. The aqueous layer was transferred to a new 2 mL tube and kept on ice. The soil pellet was re-extracted with 85 uL 5M NaCl and 0.5 mL extraction buffer, mixed, centrifuged, and the aqueous layers were combined. The combined aqueous solution was washed with chloroform:isoamyl alcohol (24:1), vortexed, and centrifuged. The new aqueous layer was transferred to a new 2 mL tube and mixed with 40% PEG/1.6M NaCl by inversion (~10 times), then incubated on ice for 2 hours. Following incubation, samples were centrifuged for 30 min. The nucleic acid pellet was washed twice with 1 mL 70% ice-cold ethanol, vortexed, and centrifuged for 10 min at 16,000 x *g* at 4°C, with supernatant decanted after each wash. The pellet was air-dried and resuspended in 50 µL TE.

16S Sequencing Library Preparation for ^{13}C amino acid Stable Isotope Probing fractions:

The rRNA 16S V4 region 515F and 806R primers [2, 3] were modified to include the Illumina platform adaptor sequences[4]. The adapter modified primer sequences (5'TCGTCGGCAGCGTCAGATGTGTATAAGAGACAGGTGYCAGCMGCCGCGGTAA-3' and 5'-GTCTCGTGGGCTCGGAGATGTGTATAAGAGACAGGGACTACNVGGGTWTCTAAT-3') were used to amplify sample DNA for library creation. DNA was amplified in 1X of Phusion

High-Fidelity Mastermix (ThermoFisher, Waltham, MA, USA), 200 nM of primers, 3% DMSO, 0.5 ug/ul of BSA, and 0.1 uL uL-1 of DNA. The following cycling conditions used: 3 minutes at 98 °C, 25 cycles of 98 °C for 10 seconds, 55 °C for 30 seconds, and 72 °C for 30 seconds, with a final extension for 10 minutes at 72 °C.

The 16S amplicons were purified with 0.8X Ampure XP magnetic beads (Beckman Coulter, Brea, CA, USA) and underwent an additional round of amplification to add Dual Nextera XT indexes (Illumina Inc., Santa Clara, CA, USA) to the amplicons. These reactions were carried out in 1X Kapa HIFI HotStart ReadyMix and 0.1 uL uL^{-1} indexes at 95 °C for 3 minutes, followed by 8 cycles of 95 °C for 30 seconds, 55 °C for 30 seconds, and 72 °C for 30 seconds, and extended at 72 °C for 5 minutes. As second round of bead purification was performed with 1.2X magnetic beads and the final libraries were pooled in equimolar ratios. The pool was denatured in NaOH and diluted to 8 pM. 15% of phiX was added to the library and the mix was run on the Illumina MiSeq Sequencer at Lawrence Livermore National Laboratory for paired end reads for 2X 250 cycles.

Analysis

For un-isotope-labeled samples, sequence reads were demultiplexed (split_libraries_fastq.py) using Qiime (v1.9.1)[5] via MacQiime v1.9.1. Sequences were then quality controlled using the dada2 framework. Briefly, sequences were length trimmed based on quality profiles, then sequences with max number of 'N' > 0 and/or maxed expected error > 2 were removed. Then paired reads were merged and collated into an OTU table. Finally, chimeras were removed and taxonomy was assigned using Silva v132 (nr) taxonomy reference database [6]. Quality controlled sequences were aligned using Muscle (v3.8.31)[7], The ASV table and taxonomy table generated in dada2, were used to create a Phyloseq (v3.7)[8]. object in R (v3.4.1) (Team, 2013) (http://www.R-project.org/). Sample libraries with less than 8,000 reads were removed from the dataset and reads in remaining libraries were normalized using DeSeq2 v1.18.1 variance stabilization[9].

For stable isotope probing samples, 16S rRNA gene sequences were amplified with the 515F[3] (GTGYCAGCMGCCGCGGTAA) and 806R[2] (GGACTACNVGGGTWTCTAAT) PCR primers, and resulting amplicons were sequenced on an Illumina MiSeq in four batches to obtain 251 bp paired-end reads. Primer sequences were identified and removed from the reads using

cutadapt[10] v4.3. Reads were filtered and processed to amplicon sequence variants (ASVs) independently for the 4 sequencing batches using DADA2[11] v1.28 and R v4.3.1. DADA2 filtering parameters for both forward and reverse reads were truncLen 200, maxEE 2, truncQ 2, and rm.phix TRUE. ASV sequence tables from the 4 batches were merged using the DADA2 mergeSequenceTables() function, then filtered for chimeric sequences using removeBimeraDenovo() with method = "consensus". The ASV table was further filtered to remove ASVs not found at least twice in at least two separate samples. ASV sequences were aligned with mafft[12] v7.505 and a tree was generated using fasttree [13] v2.1.11. Taxonomy was assigned using RDP[14] with training set 18.

Identified Fe Reducers and Methanogens from Literature-Reported Species:

Taxa	Function	DOI
Anaeromyxobacter dehalogenans	Fe reduction	10.1128/AEM.69.5.2712-2718.2003
Bacillus infernus	Fe reduction	10.1099/00207713-45-3-441
Desulfitobacterium frappieri	Fe reduction	10.1080/01490450303884
Desulfobulbus propionicus	Fe reduction	10.4056/sigs.1613929
Desulfosporosinus meridiei	Fe reduction	10.1128/JB.01392-12
Desulfovibrio profundus	Fe reduction	10.1099/00207713-47-2-515
Geobacter sp.	Fe reduction	10.1128/MRA.00913-21
Geothrix fermentans	Fe reduction	10.1128/AEM.01460-12
Klebsiella sp.	Fe reduction	10.1016/j.colsurfa.2010.11.061
Methanobacterium sp.	Methanogenesis	10.1128/jb.109.2.707-713.1972
Methanosarcina sp.	Methanogenesis	10.1128/AEM.00731-21

SECTION 3

ACETATE ANALYSES BY NMR

For the collected aqueous phase samples at the end of the last (third) oxic interval for the treatments Ox-72, Ox-24, Ox-8, and Pre-Conditioning, metabolite analyses (acetate) were performed by Nuclear magnetic resonance spectroscopy (NMR). The aqueous extracts (180 uL) were diluted with perdeuterated sodium 4,4-dimethyl-4-silapentane-1-sulfonate (DSS-d6) in D_2O (5 mM, 20 uL). The resulting 0.5 mM dDSS (4,4-dimethyl-4-silapentane-1-sulfonic acid) in 10% D_2O/ 90% H_2O were used as an internal calibrant. The Varian Direct Drive 600-MHz NMR spectrometer (with a 5-mm triple resonance cold probe) was used to collect the NMR spectra for all samples. The samples were analyzed in 3 mm NMR tubes, with the temperature regulated at 298 K. Chemical shifts were due to the ^1H or ^{13}C methyl signals, in DSS-d6 at 0 ppm. The 90° ^1H pulse was calibrated before the measurement of each sample. The spectra for the one-dimensional ^1H were obtained with the Varian pulse sequence, containing a spectral width of 12 ppm and 512 transients. The acquisition time was 4 s, followed by a relaxation delay of 1.5 s during which pre-saturation of the water signal was applied, and the NOESY mixing time was 100 ms. Time domain free induction decays (57472 total points) were zero filled to 131072 total points prior to Fourier transform.

The 1D ^1H spectra were manually phased, baseline corrected, assigned metabolite identifications, and computed using Chenomx NMR Suite 8.3. The identification for each metabolite was performed by matching the chemical shift, J-coupling and intensity of experimental signals against compound signals in the Chenomx, Human Metabolome Database (HMDB) and custom in-house databases. Spectra were quantified based on intensity relative to the 0.5mM DSS-d6 (CAS-no 284664-85-3) internal standard. Spike-in amendments of acetate (ca. ~20 uM) were made into 4 representative samples to further confirm this metabolite identification.

SECTION 4

FIGURES AND TABLES

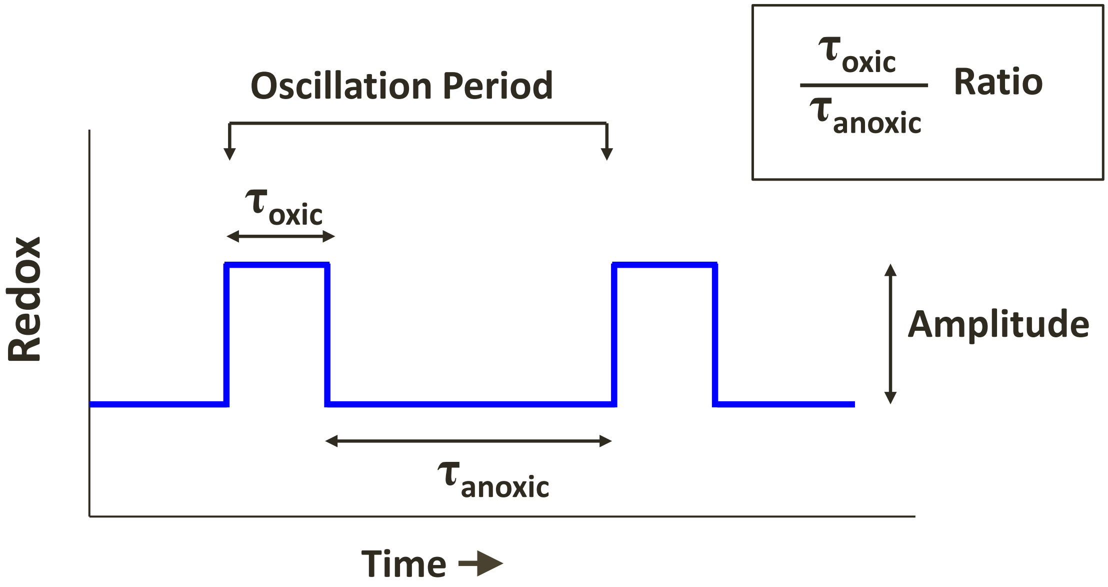

Figure S1 Components of a hypothetical redox oscillation cycle, proposed in Barcellos et al. (2018) [15].

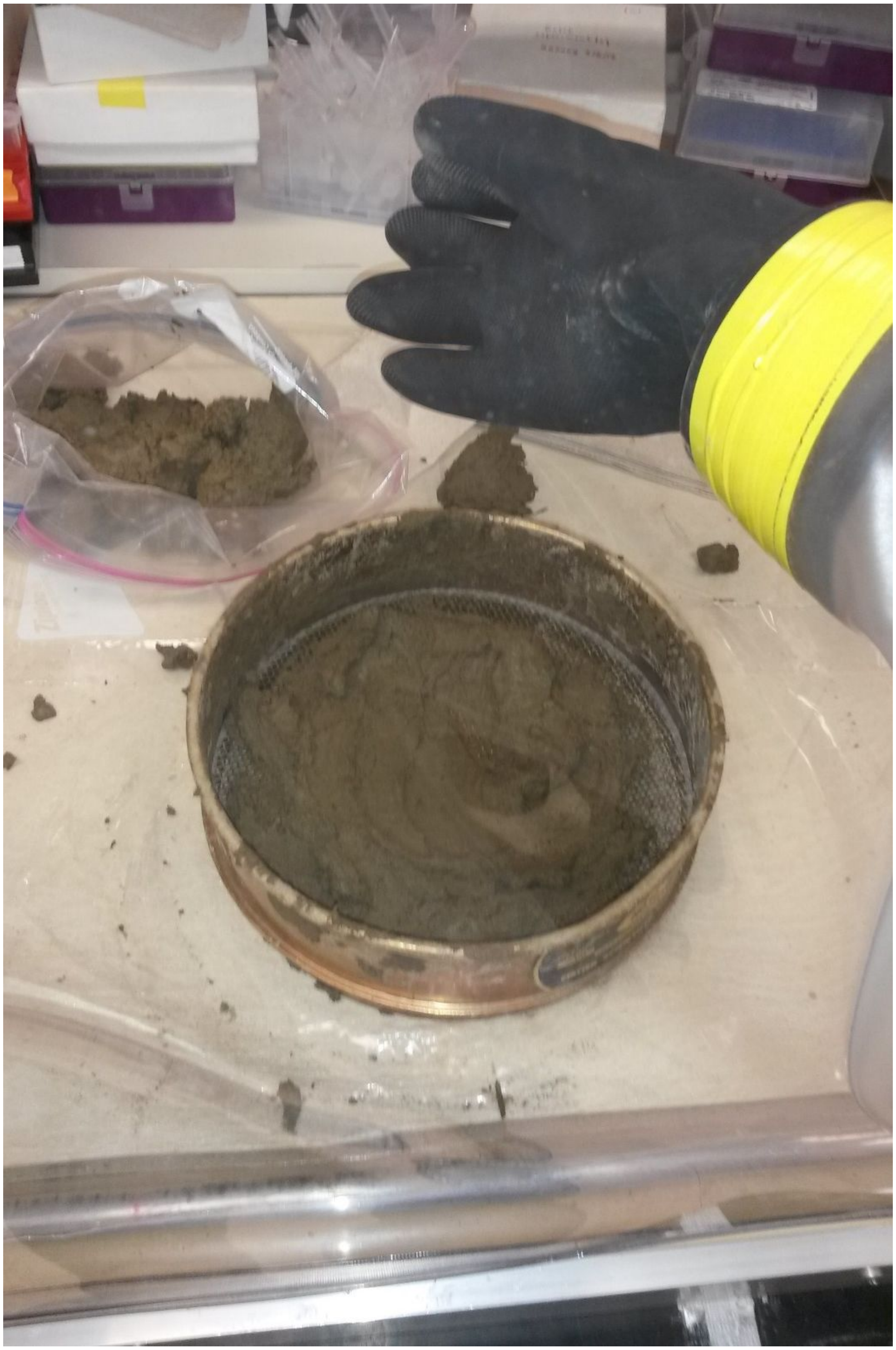

Figure S2. Soil sample sieved at 2 mm inside a 95%:5%:0% (N_2:H_2:O_2) glovebox Coy chamber. Soils were obtained from the Luquillo Experimental Forest, Bisley watershed (valley topographic position), Puerto Rico.

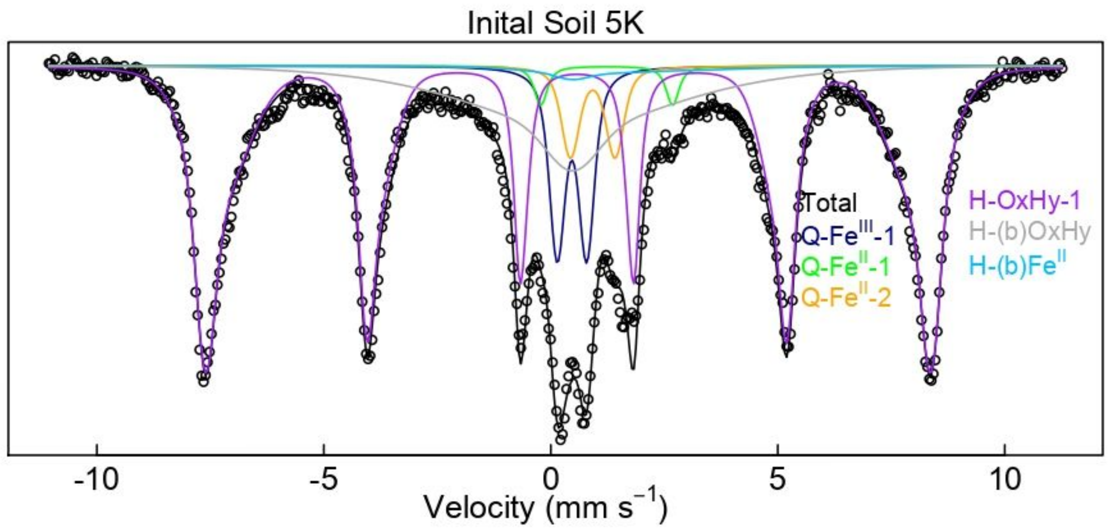

Figure S3. Mössbauer spectra (5 K) for the soil at the beginning of the redox oscillation experiment (initial soil). For each spectrum, the black line corresponds to the total calculated fit, through the discrete data points. Detailed fitting parameters are provided in Table S3. The resolved spectral components and assignments are: (1) **Q-Fe^{III}-1,** the deep central doublet (blue line) corresponding to Fe^{III} in aluminosilicates or organic matter; (2) **QFe^{II}-1**, the wide ferrous doublet corresponding to adsorbed Fe^{II} or Fe^{II} in clays/organic matter (green line); (3) **Q-Fe^{II}-2** the narrow ferrous doublet corresponding to Fe^{II} in ilmenite (brown line); (4) **HFD-OxHy-1**, the dominant sextet (purple line) corresponding to Fe^{III}-oxyhydroxides that are magnetically ordered; (5) **HFD-(b)OxHy** the collapsed 'sextet' corresponding to Fe^{III} oxyhydroxides near their blocking temperature; and (6) H-(b)Fe^{II} partially magnetically ordered Fe^{II} phase.

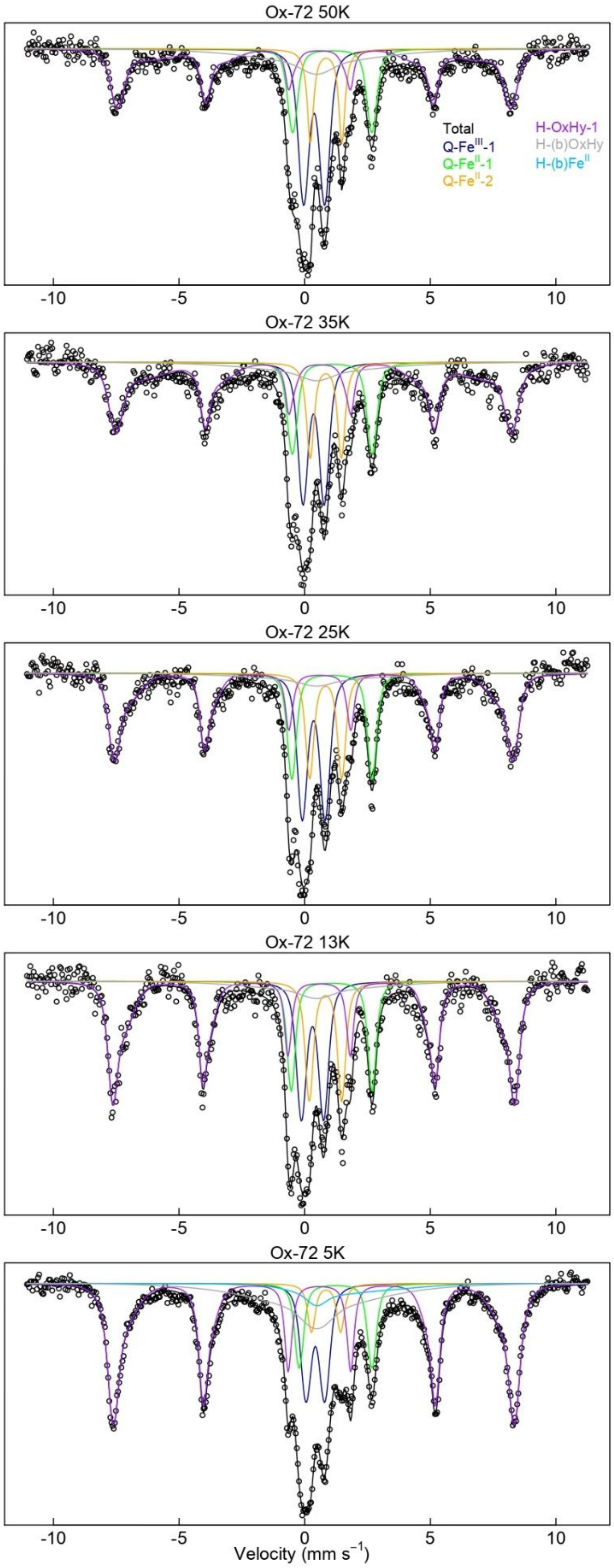

Figure S4. Mössbauer spectra (50 K, 35 K, 25 K, 13 K, 5 K) for soils collected at the end of the last (third) oxic interval, for the redox oscillation treatment **Ox-72** (oxygenation for 72 h). For each spectrum, the black line corresponds to the total calculated fit, through the discrete data points. Detailed fitting parameters are provided in the Supplementary Material (Table S4). The resolved spectral components and assignments are: (1) **Q-Fe^{III}-1,** the deep central doublet (blue line) corresponding to Fe^{III} in aluminosilicates or organic matter; (2) **QFe^{II}-1**, the wide ferrous doublet corresponding to adsorbed Fe^{II} or Fe^{II} in clays/organic matter (green line); (3) **Q-Fe^{II}-2** the narrow ferrous doublet corresponding to Fe^{II} in ilmenite (brown line); (4) **HFD-OxHy-1**, the dominant sextet (purple line) corresponding to Fe^{III}-oxyhydroxides that are magnetically ordered; (5) **HFD-(b)OxHy** the collapsed 'sextet' corresponding to Fe^{III} oxyhyroxides near their blocking temperature; and (6) H-(b)Fe^{II} partially magnetically ordered Fe^{II} phase.

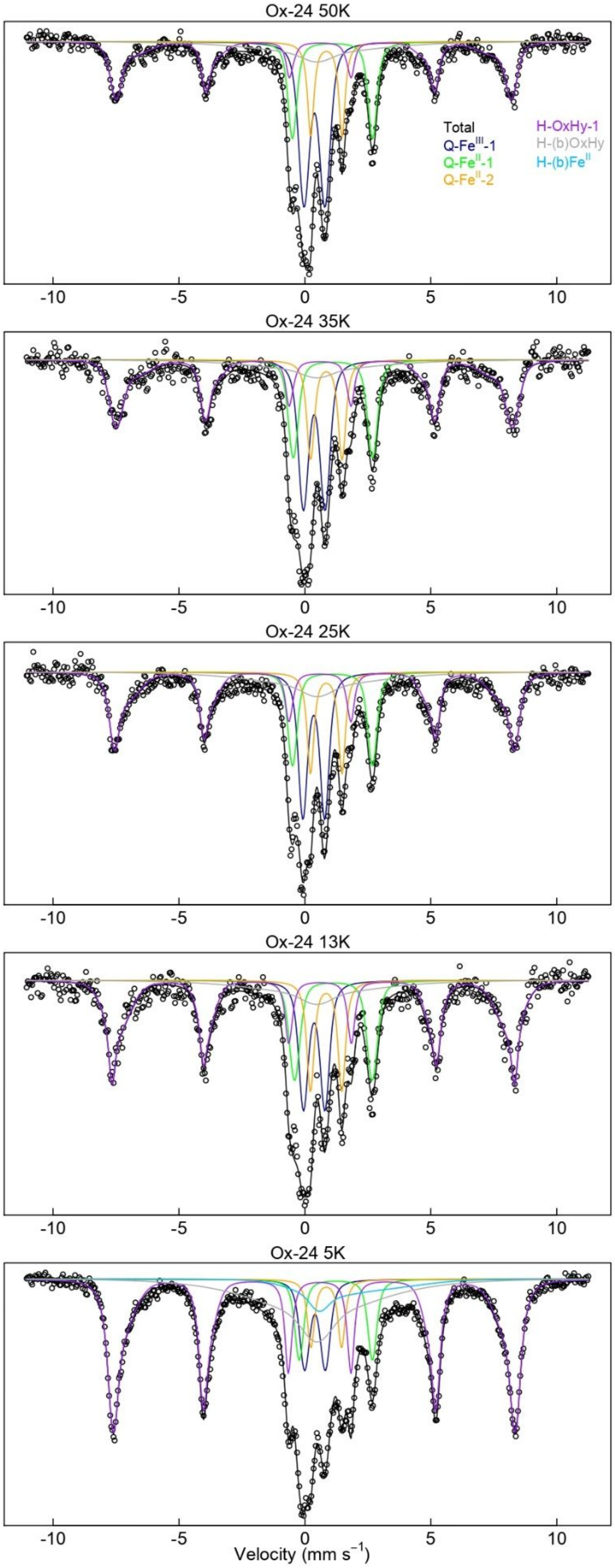

Figure S5. Mössbauer spectra (50 K, 35 K, 25 K, 13 K, 5 K) for soils collected at the end of the last (third) oxic interval, for the redox oscillation treatment **Ox-24** (oxygenation for 24 h). For each spectrum, the black line corresponds to the total calculated fit, through the discrete data points. Detailed fitting parameters are provided in the Supplementary Material (Table S5). The resolved spectral components and assignments are: (1) **Q-Fe^{III}-1,** the deep central doublet (blue line) corresponding to Fe^{III} in aluminosilicates or organic matter; (2) **QFe^{II}-1**, the wide ferrous doublet corresponding to adsorbed Fe^{II} or Fe^{II} in clays/organic matter (green line); (3) **Q-Fe^{II}-2** the narrow ferrous doublet corresponding to Fe^{II} in ilmenite (brown line); (4) **HFD-OxHy-1**, the dominant sextet (purple line) corresponding to Fe^{III}-oxyhydroxides that are magnetically ordered; (5) **HFD-(b)OxHy** the collapsed 'sextet' corresponding to Fe^{III} oxyhyroxides near their blocking temperature; and (6) H-(b)Fe^{II} partially magnetically ordered Fe^{II} phase.

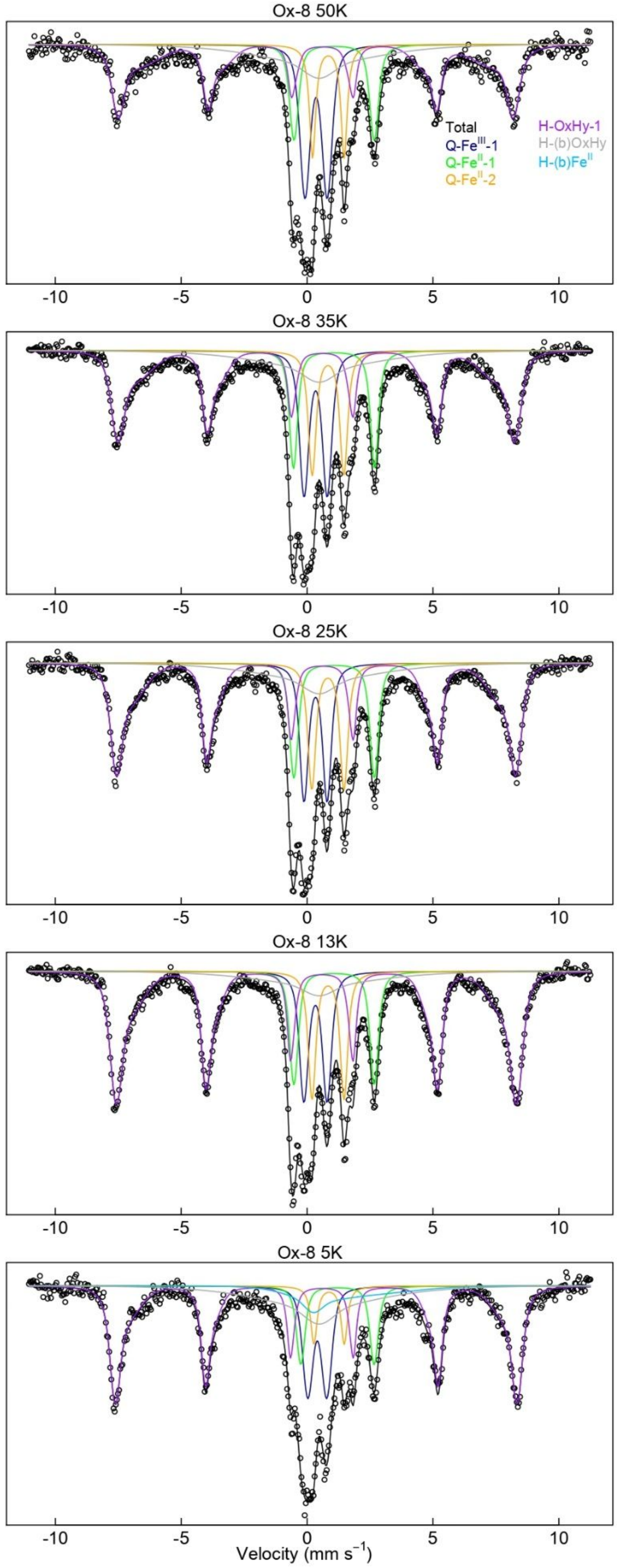

Figure S6. Mössbauer spectra (50 K, 35 K, 25 K, 13 K, 5 K) for soils collected at the end of the last (third) oxic interval, for the redox oscillation treatment **Ox-8** (oxygenation for 8 h). For each spectrum, the black line corresponds to the total calculated fit, through the discrete data points. Detailed fitting parameters are provided in the Supplementary Material (Table S6). The resolved spectral components and assignments are: (1) **Q-Fe^{III}-1,** the deep central doublet (blue line) corresponding to Fe^{III} in aluminosilicates or organic matter; (2) **QFe^{II}-1**, the wide ferrous doublet corresponding to adsorbed Fe^{II} or Fe^{II} in clays/organic matter (green line); (3) **Q-Fe^{II}-2** the narrow ferrous doublet corresponding to Fe^{II} in ilmenite (brown line); (4) **HFD-OxHy-1**, the dominant sextet (purple line) corresponding to Fe^{III}-oxyhydroxides that are magnetically ordered; (5) **HFD-(b)OxHy** the collapsed 'sextet' corresponding to Fe^{III} oxyhyroxides near their blocking temperature; and (6) H-(b)Fe^{II} partially magnetically ordered Fe^{II} phase.

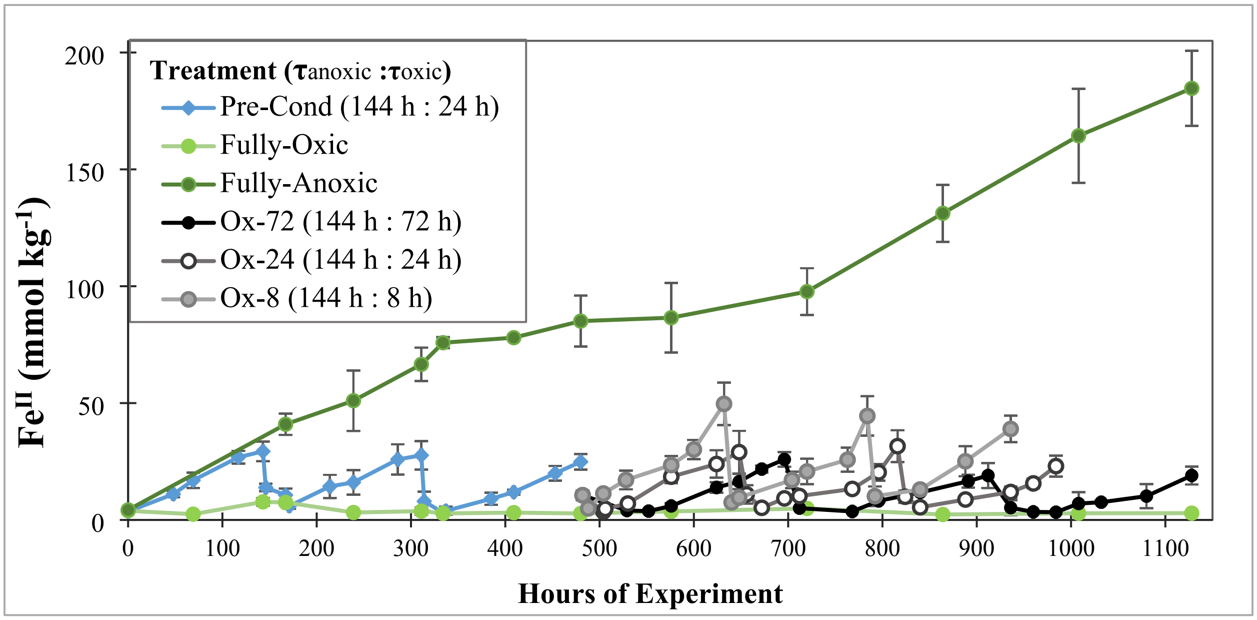

Figure S7. Fe^{II} dynamics (mean ± 1 standard deviation) for all treatments including fully-oxic and fully-anoxic controls. In the fully-anoxic treatment (non-fluctuating), Fe^{II} concentrations increased continuously and peaked at 185 ± 16 mmol kg^{-1} at the end (equal to approximately half the soil's Short-Range Order (SRO) Fe^{III} content of 439 ± 7 mmol kg^{-1}.

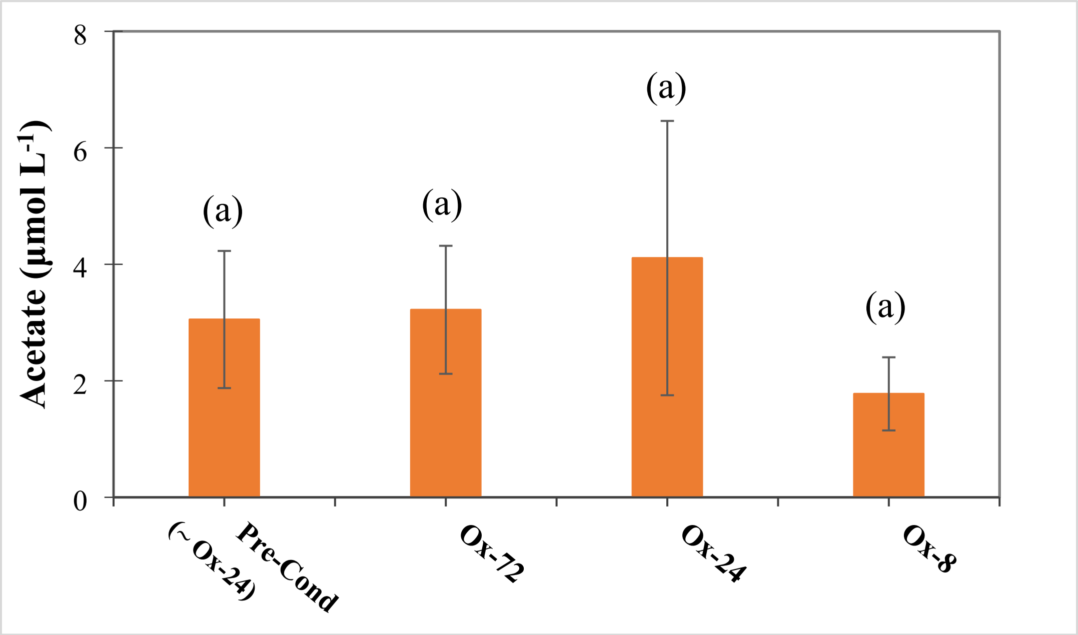

Figure S8. Acetate (µmol L^{-1}) in the aqueous phase of the reactors, sampled in the last (third) oxidation event for the treatments (Ox-72, Ox-24, and Ox-8) and for the pre-conditioning. Analyses performed in Nuclear magnetic resonance spectroscopy (NMR) for liquid samples. Different lowercase letters in parentheses indicate significant differences at the 5% probability level (ANOVA with Tukey HSD test) for each metabolite. The error bars indicate a ± 1 standard deviation.

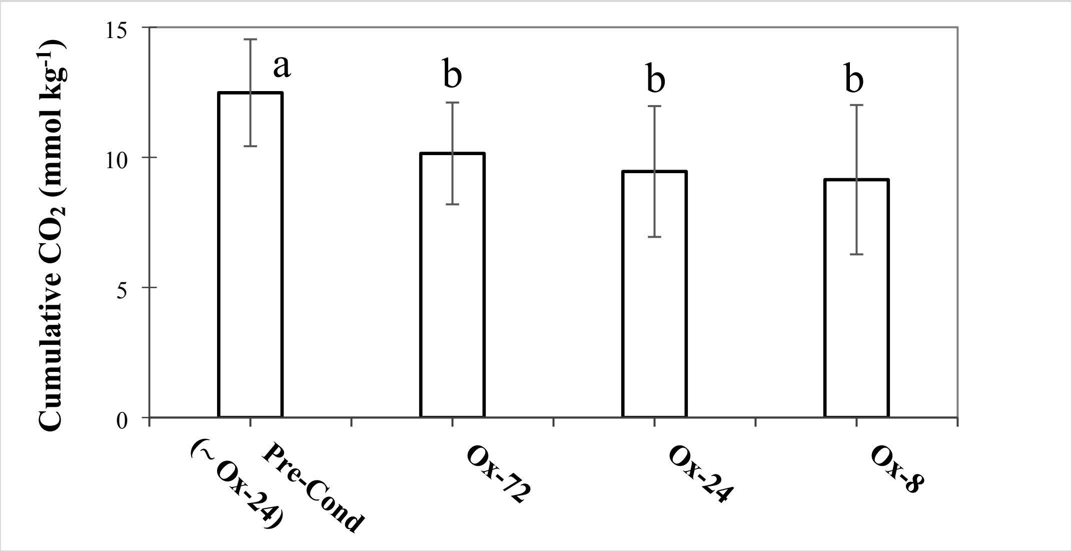

Figure S9. Cumulative CO_2 from headspace of incubated soils from Luquillo Experimental Forest along the τ_{anoxic} intervals for each treatment. Lowercase letters in parentheses (a and b) indicate significant differences at the 5% probability level. The error bars indicate a ± 1 standard deviation.

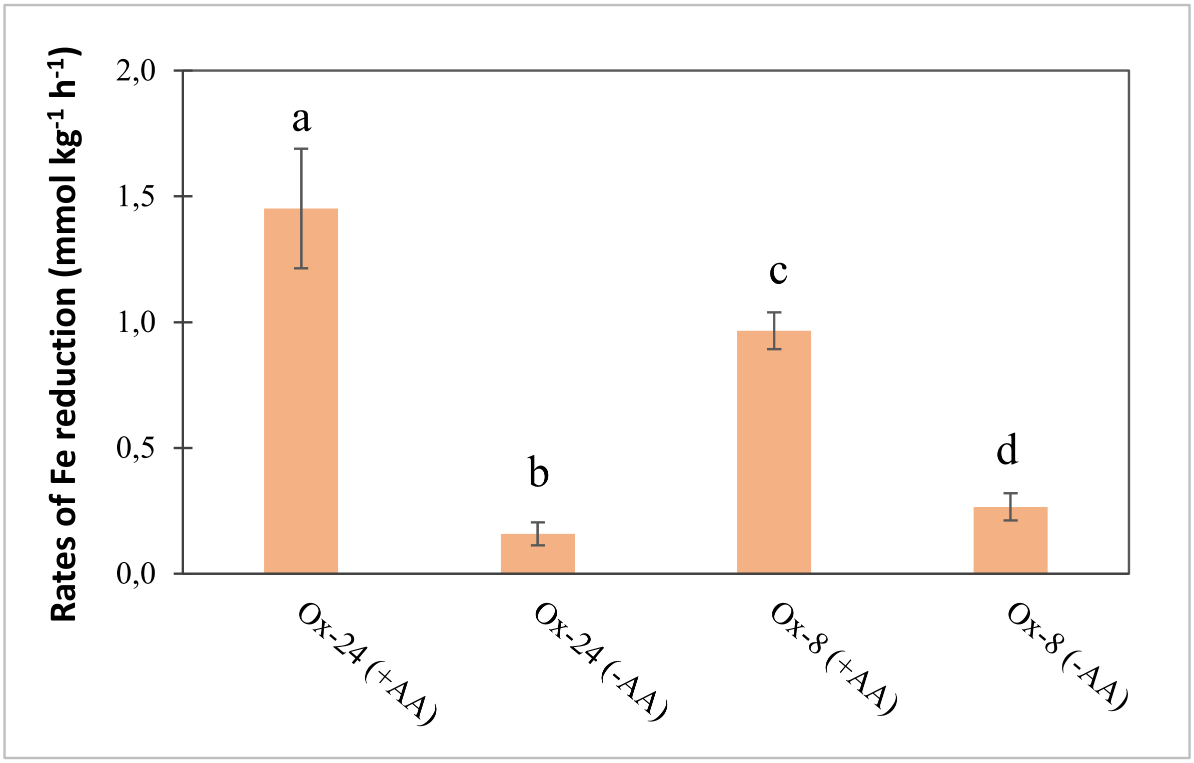

Figure S10. Rates of Fe reduction (mean ± 1 standard deviation), in the last anoxic interval, comparing Ox-24 and Ox-8 treatments with (+AA) and without (-AA) the amendment of amino acids. Lowercase letters in parentheses (a to d) indicate significant differences at the 5% probability level.

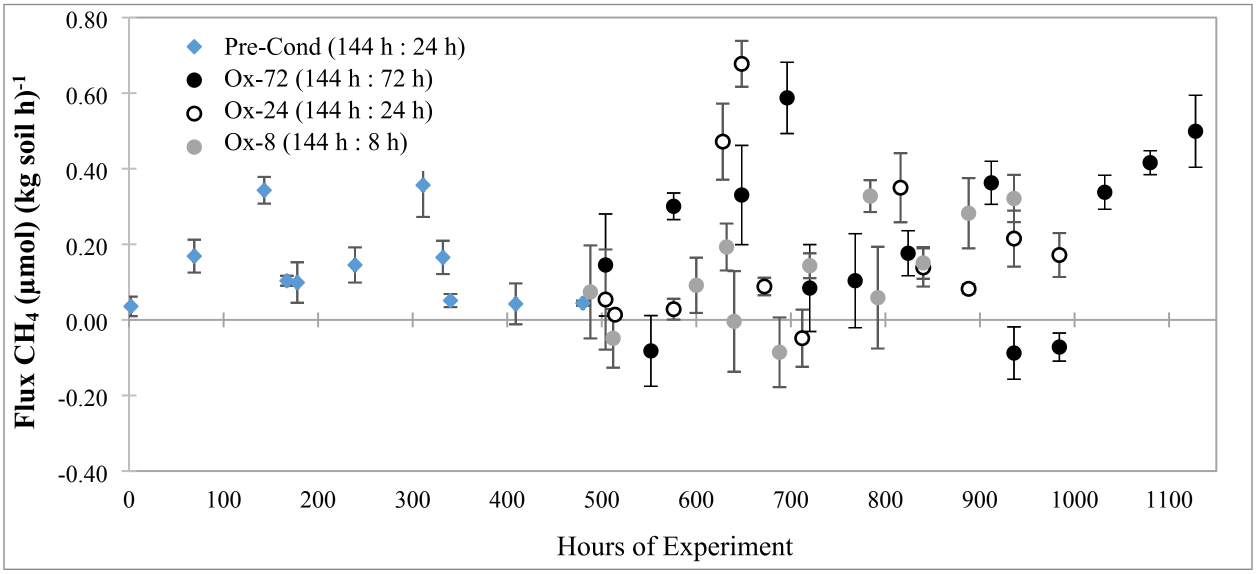
Flux CH4 (µmol) (kg soil h)-1
Hours of Experiment
Pre-Cond (144 h : 24 h)
Ox-72 (144 h : 72 h)
Ox-24 (144 h : 24 h)
Ox-8 (144 h : 8 h)
0.80
0.60
0.40
0.20
0.00
-0.20
-0.40
0
100
200
300
400
500
600
700
800
900
1000
1100

(a)

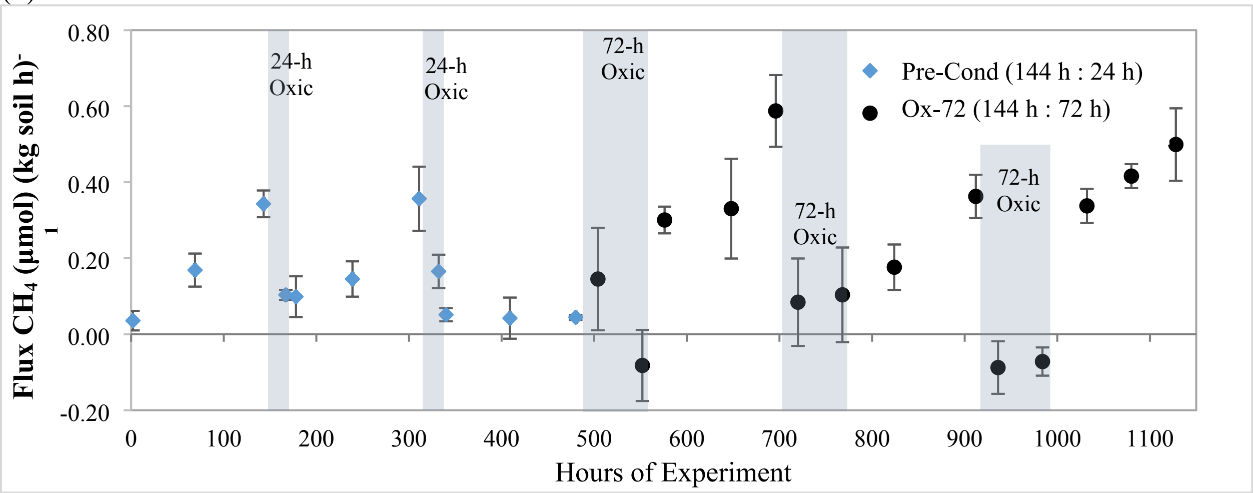
Flux CH4 (µmol) (kg soil h)-1
Hours of Experiment
24-h Oxic
24-h Oxic
72-h Oxic
72-h Oxic
72-h Oxic
Pre-Cond (144 h : 24 h)
Ox-72 (144 h : 72 h)
0.80
0.60
0.40
0.20
0.00
-0.20
0
100
200
300
400
500
600
700
800
900
1000
1100

(b)

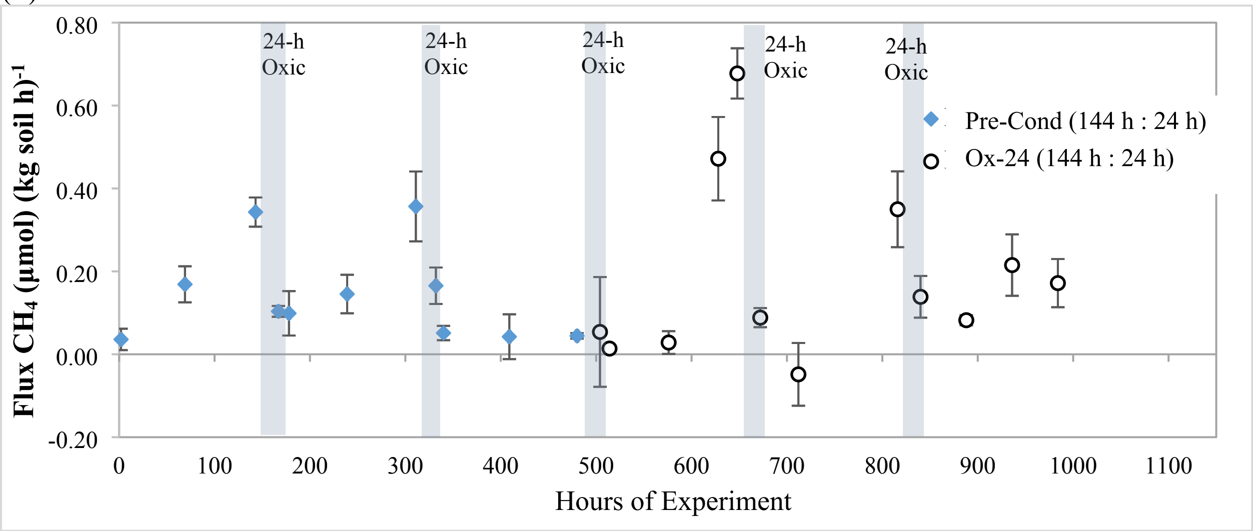
Flux CH4 (µmol) (kg soil h)-1
Hours of Experiment
24-h Oxic
24-h Oxic
24-h Oxic
24-h Oxic
24-h Oxic
Pre-Cond (144 h : 24 h)
Ox-24 (144 h : 24 h)
0.80
0.60
0.40
0.20
0.00
-0.20
0
100
200
300
400
500
600
700
800
900
1000
1100

(c)

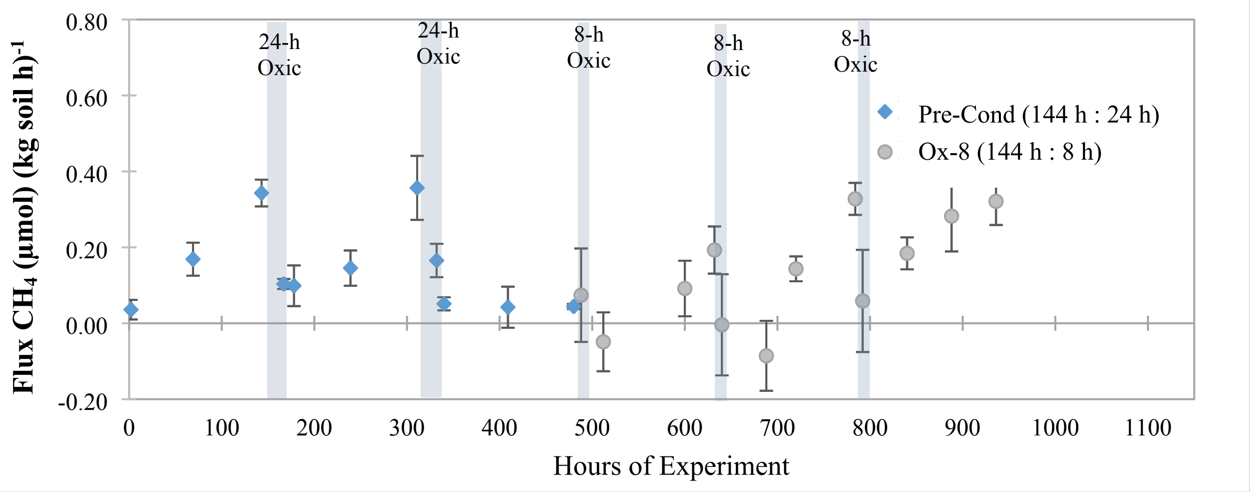

(d)

Figure S11. Instantaneous CH_4 flux from headspace of incubated soils from Luquillo Experimental Forest for all treatments together (a) and over the different treatments (b) to (d). The error bars indicate a ± 1 standard deviation.

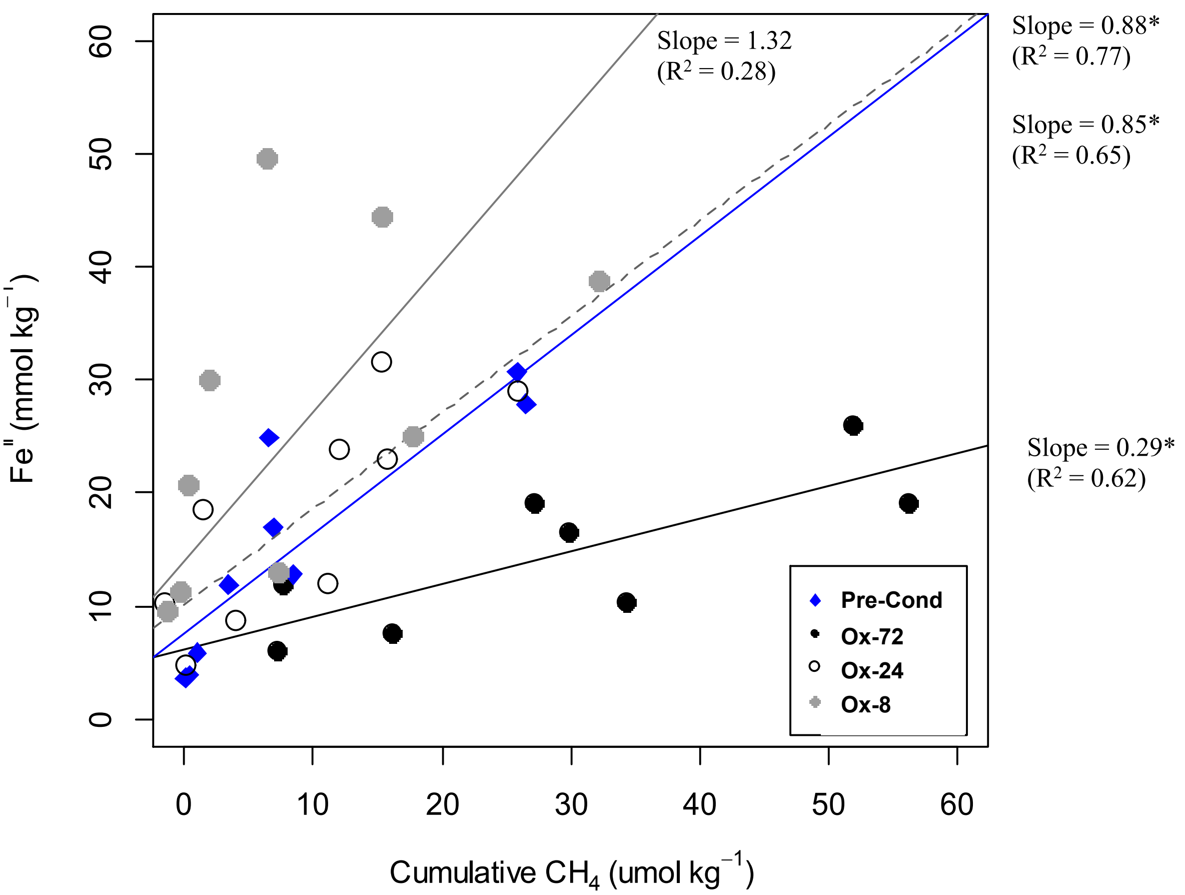

Figure S12. Linear regressions for Fe^{II} concentrations vs cumulative CH_4, under anoxic conditions only, for all treatments. Asterisks represent slopes that are significant at 5% probably for the linear models.

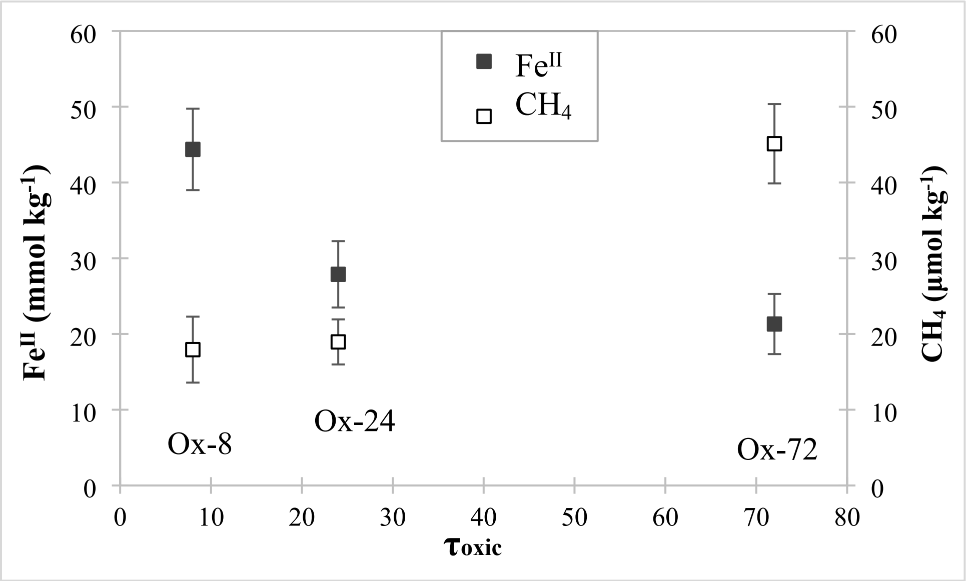

Figure S13. Soil Fe^{II} concentrations and cumulative CH_4 during anoxic conditions, comparing different previous exposed τ_{oxic} of 8, 24, and 72 h treatments.

Table S1. Total elemental analysis for initial native soil sample (no treatments or chemicals added). Soils from Bisley watershed, Puerto Rico.

Fe	Al	Si	Ca	Mg	Na	K	Ti	Mn	P
----------- mmol kg^{-1} --------			------------------------------------ % ------------------------------------						
943	2501	13636	1.54	0.65	1.00	0.43	0.39	0.14	0.035

Short-range-ordered Iron phases by citrate-ascorbate extration[16] (SRO-Fe^{III}) = 439 ± 7 mmol kg^{-1}.

Table S2. Dissolved oxygen (DO) concentrations exposed to anoxic conditions, from soil slurries previously exposed to oxic conditions. Zero time is still under oxic condition, measurements taken right before placing inside the anoxic chamber. Soils were collected from the Luquillo Experimental Forest, Bisley watershed, Puerto Rico.

**Time exposed to anoxic conditions**	**Dissolved Oxygen (DO)**
Hours	*µg L^{-1}*
0	9800 ± 100
0.05	2967 ± 208
0.1	1068 ± 46
2	241 ± 13
5	119 ± 9

Table S3. Mössbauer spectral parameters for the soil at the beginning of the redox oscillation experiment (initial soil). The spectra are presented in Figure S3.

Sample	Phase	Area	$\overline{CS}$ or δ_0	$\overline{\epsilon}$	P	Δ or H	σ	$\overline{QS}$ or $\overline{H}$	χ^2_ν
Inital Soil 5K	QSD site 1	9.69(44)	0.4648(84)	n/a	100*	0.650(13)	0.195(20)	0.65	2.08
	QSD site 2	1.78(36)	1.230(28)	n/a	100*	2.903(56)	0.11(12)	2.9	
	QSD site 3	5.19(49)	0.923(15)	n/a	100*	0.983(27)	0.252(47)	0.98	
	HFD site 1	61.3(14)	0.4798(29)	-0.0970(29)	42.7068*	49.572(38)	0.923(65)	48.35	
					57.3(24)	47.45(16)	3.67(12)		
	HFD site 2	20.2(12)	0.48*	0*	100*	0*	20*	15.96	
(BG = 4.4 MC/ch)	HFD site 3	1.8(15)	1.47*	1*	100*	0*	11.5*	9.18	

BG = background level, in mega-counts per channel (MC/ch).

$Phase$ = assigned spectral component, as described in the text.

$\overline{CS}$ **or** δ_0 = the center shift of a Gaussian component in the quadrupole splitting distribution (QSD) of the hyperfine field distribution (HFD) of a given spectral component, given in mm s^{-1} .

$\overline{QS}$ = the center shift of a Gaussian component in the quadrupole splitting distribution (QSD) of the hyperfine field distribution (HFD) of a given spectral component, given in mm s^{-1} .

σ = the Gaussian standard deviation width of a given Gaussian component of a given QSD or HFD.

P = the weight factor (%) for a given Gaussian component in a given QSD or HFD.

H and $\overline{H}$ = the average magnitude of the hyperfine field (expressed as an excited state Zeeman splitting, in Torr (T), in a given HFD of a given sextet spectral component, or all components, respectively.

Δ = the average magnitude of the quadrupole splitting in a given QSD of a given doublet spectral component, given in mm s^{-1}.

$\overline{\epsilon}$ = the average magnitude of the slave distribution of quadrupole shifts (ϵ) associated to a given HFD of a given sextet spectral component, given in mm s^{-1}.

χ^2_ν = the reduced chi-squared value for the fit: chi-squared (χ^2) divided by the number of degrees of freedom (ν). It has an ideal value of 1 for a correct model.

All fits performed using the Voigt-based fitting method of Rancourt and Ping (1991) with the Recoil^{TM} software.

All fitting and calculated parameters are as defined in by Rancourt and Ping (1991).

All $\delta - 1$ couplings between CS and H (or DELTA) were taken to be zero.

All line-1 to line-2 area ratios in all (distributed) elemental doublets were taken to be 1.

All line-2/line-3 and line-1/line-3 area ratios in all (distributed and symmetric) elemental sextets were taken to be 2 and 3, respectively.

All $\epsilon - 1$ couplings between epsilon and H (in a HFD) are taken to be 0.

All Lorentzian half widths at half maximum (HWHM) are set at 0.1425 mm s^{-1} as measured on Fe foil standards on the instrument.

All center shifts ($\overline{CS}$ or δ_0) are given with respect to the CS of metallic Fe at 295K.

Table S4. Mössbauer spectral parameters for soils collected at the end of the last (third) oxic interval, for the redox oscillation treatment **Ox-72** (oxygenation for 72 h). The spectra are presented in Figure S4.

Sample	Phase	Area	$\overline{CS}$ or δ_0	$\bar{\epsilon}$	P	Δ or H	σ	$\overline{QS}$ or $\overline{H}$	χ^2_ν
Ox-8 50K	QSD site 1	21.04(98)	0.3573(91)	n/a	100*	0.882(15)	0.261(22)	0.88	1.11
	QSD site 2	12.31(71)	1.074(11)	n/a	100*	3.183(23)	0.206(33)	3.18	
	QSD site 3	11.21(72)	0.8449(79)	n/a	100*	1.252(16)	0.053(57)	1.25	
	HFD site 1	39.8(14)	0.476(11)	-0.132(11)	47.6378*	48.66(11)	1.03(18)	46.17	
					52.4(62)	43.90(94)	5.10(54)		
(BG = 1.2 MC/ch)	HFD site 2	15.7(20)	0.48*	0*	100*	0*	20*	15.96	
Ox-8 35K	QSD site 1	17.29(67)	0.3404(68)	n/a	100*	0.919(11)	0.222(17)	0.92	1.17
	QSD site 2	12.79(49)	1.0696(65)	n/a	100*	3.219(13)	0.159(21)	3.22	
	QSD site 3	13.13(58)	0.8311(60)	n/a	100*	1.255(12)	0.147(21)	1.25	
	HFD site 1	43.3(12)	0.4790(64)	-0.1175(64)	54.4397*	48.890(66)	1.14(13)	46.85	
					45.6(76)	44.41(81)	3.90(54)		
(BG = 1.3 MC/ch)	HFD site 2	13.5(15)	0.48*	0*	100*	0*	20*	15.96	
Ox-8 25K	QSD site 1	14.95(66)	0.3369(78)	n/a	100*	0.916(14)	0.187(22)	0.92	1.02
	QSD site 2	12.82(54)	1.0746(80)	n/a	100*	3.195(16)	0.193(24)	3.19	
	QSD site 3	13.18(60)	0.8264(71)	n/a	100*	1.277(14)	0.161(24)	1.28	
	HFD site 1	46.4(11)	0.4780(60)	-0.1189(60)	52.3824*	49.202(66)	0.94(13)	47.52	
					47.6(69)	45.68(63)	3.33(36)		
(BG = 1 MC/ch)	HFD site 2	12.7(15)	0.48*	0*	100*	0*	20*	15.96	
Ox-8 13K	QSD site 1	14.08(59)	0.3342(79)	n/a	100*	0.920(14)	0.206(21)	0.92	1.33
	QSD site 2	12.10(47)	1.0739(73)	n/a	100*	3.193(15)	0.187(23)	3.19	
	QSD site 3	12.44(52)	0.8318(63)	n/a	100*	1.272(12)	0.139(22)	1.27	
	HFD site 1	51.6(10)	0.4770(46)	-0.1074(46)	57.0762*	49.328(54)	1.02(10)	47.9	
					42.9(60)	46.00(58)	3.35(31)		
(BG = 1.4 MC/ch)	HFD site 2	9.8(14)	0.48*	0*	100*	0*	20*	15.96	
Ox-8 5K	QSD site 1	14.8(18)	0.404(19)	n/a	100*	0.754(30)	0.272(48)	0.75	0.63
	QSD site 2	9.3(14)	1.206(17)	n/a	100*	2.912(35)	0.185(64)	2.91	
	QSD site 3	5.6(12)	0.872(24)	n/a	100*	1.205(51)	0.06(14)	1.21	
	HFD site 1	45.0(42)	0.4737(84)	-0.1122(84)	58.36*	49.534(97)	0.81(18)	48.38	
					42(10)	46.76(96)	3.14(53)		
	HFD site 2	17.0(54)	0.48*	0*	100*	0*	20*	15.96	
(BG = 3.3 MC/ch)	HFD site 3	8.3(57)	1.24(25)	1*	100*	0*	11.5(68)	9.16	

BG = background level, in mega-counts per channel (MC/ch).

$Phase$ = assigned spectral component, as described in the text.

$\overline{CS}$ **or** δ_0 = the center shift of a Gaussian component in the quadrupole splitting distribution (QSD) of the hyperfine field distribution (HFD) of a given spectral component, given in mm s^{-1} .

$\overline{QS}$ = the center shift of a Gaussian component in the quadrupole splitting distribution (QSD) of the hyperfine field distribution (HFD) of a given spectral component, given in mm s^{-1} .

σ = the Gaussian standard deviation width of a given Gaussian component of a given QSD or HFD.

P = the weight factor (%) for a given Gaussian component in a given QSD or HFD.

H and $\overline{H}$ = the average magnitude of the hyperfine field (expressed as an excited state Zeeman splitting, in Torr (T), in a given HFD of a given sextet spectral component, or all components, respectively.

Δ = the average magnitude of the quadrupole splitting in a given QSD of a given doublet spectral component, given in mm s^{-1}.

$\bar{\epsilon}$ = the average magnitude of the slave distribution of quadrupole shifts (ϵ) associated to a given HFD of a given sextet spectral component, given in mm s^{-1}.

χ^2_ν = the reduced chi-squared value for the fit: chi-squared (χ^2) divided by the number of degrees of freedom (ν). It has an ideal value of 1 for a correct model.

All fits performed using the Voigt-based fitting method of Rancourt and Ping (1991) with the Recoil^{TM} software.

All fitting and calculated parameters are as defined in by Rancourt and Ping (1991).

All $\delta - 1$ couplings between CS and H (or DELTA) were taken to be zero.

All line-1 to line-2 area ratios in all (distributed) elemental doublets were taken to be 1.

All line-2/line-3 and line-1/line-3 area ratios in all (distributed and symmetric) elemental sextets were taken to be 2 and 3, respectively.

All $\epsilon - 1$ couplings between epsilon and H (in a HFD) are taken to be 0.

All Lorentzian half widths at half maximum (HWHM) are set at 0.1425 mm s^{-1} as measured on Fe foil standards on the instrument.

All center shifts ($\overline{CS}$ or δ_0) are given with respect to the CS of metallic Fe at 295K.

Table S5. Mössbauer spectral parameters for soils collected at the end of the last (third) oxic interval, for the redox oscillation treatment **Ox-24** (oxygenation for 24 h). The spectra are presented in Figure S5.

Sample	Phase	Area	$\overline{CS}$ or δ_0	$\overline{\epsilon}$	P	Δ or H	σ	$\overline{QS}$ or $\overline{H}$	χ^2_ν
Ox-24 50K	QSD site 1	29.8(15)	0.3817(97)	n/a	100*	0.833(16)	0.290(23)	0.83	0.67
	QSD site 2	15.7(10)	1.098(12)	n/a	100*	3.184(24)	0.204(35)	3.18	
	QSD site 3	11.91(98)	0.854(10)	n/a	100*	1.243(21)	0.053(74)	1.24	
	HFD site 1	30.7(17)	0.481(14)	-0.130(14)	68.0536*	48.70(13)	0.99(28)	47.13	
					32(19)	43.8(28)	3.5(21)		
(BG = 5.5 MC/ch)	HFD site 2	11.9(27)	0.48*	0*	100*	0*	20*	15.96	
Ox-24 35K	QSD site 1	24.1(19)	0.372(15)	n/a	100*	0.862(24)	0.263(37)	0.86	0.66
	QSD site 2	16.1(15)	1.114(18)	n/a	100*	3.146(37)	0.257(50)	3.15	
	QSD site 3	13.2(15)	0.852(16)	n/a	100*	1.249(33)	0.142(57)	1.25	
	HFD site 1	36.7(24)	0.491(18)	-0.113(18)	72.2531*	48.82(25)	1.27(34)	47.36	
					28(25)	43.5(37)	3.1(29)		
(BG = 2.9 MC/ch)	HFD site 2	9.8(39)	0.48*	0*	100*	0*	20*	15.96	
Ox-24 25K	QSD site 1	21.4(14)	0.354(12)	n/a	100*	0.867(21)	0.207(31)	0.87	0.66
	QSD site 2	14.4(11)	1.095(16)	n/a	100*	3.170(32)	0.221(45)	3.17	
	QSD site 3	12.5(12)	0.850(13)	n/a	100*	1.247(25)	0.098(57)	1.25	
	HFD site 1	38.5(20)	0.490(13)	-0.107(13)	49.1033*	49.29(14)	0.70(28)	47.78	
					51(13)	46.3(11)	3.07(63)		
(BG = 3.9 MC/ch)	HFD site 2	13.3(30)	0.49*	0*	100*	0*	20*	15.96	
Ox-24 13K	QSD site 1	17.4(16)	0.370(18)	n/a	100*	0.850(30)	0.226(47)	0.85	0.66
	QSD site 2	15.2(14)	1.133(20)	n/a	100*	3.081(41)	0.282(55)	3.08	
	QSD site 3	12.8(14)	0.845(16)	n/a	100*	1.243(32)	0.129(59)	1.24	
	HFD site 1	43.1(24)	0.470(13)	-0.133(13)	31.5236*	49.58(15)	0.25(46)	48.58	
					68.5(86)	48.12(45)	2.89(41)		
(BG = 2.8 MC/ch)	HFD site 2	11.6(36)	0.48*	0*	100*	0*	20*	15.96	
Ox-24 5K	QSD site 1	9.41(78)	0.397(13)	n/a	100*	0.822(31)	0.252(45)	0.82	0.94
	QSD site 2	7.99(65)	1.226(12)	n/a	100*	2.902(24)	0.205(39)	2.9	
	QSD site 3	5.64(57)	0.846(14)	n/a	100*	1.217(26)	0.108(52)	1.22	
	HFD site 1	46.5(20)	0.4766(41)	-0.1069(41)	52.6774*	49.522(53)	0.756(97)	48.51	
					47.3(51)	47.38(35)	2.88(20)		
	HFD site 2	22.4(22)	0.48*	0*	100*	0*	20*	15.96	
(BG = 1.9 MC/ch)	HFD site 3	8.0(27)	1.56(19)	1*	100*	0*	11.3(33)	9.04	

BG = background level, in mega-counts per channel (MC/ch).

$Phase$ = assigned spectral component, as described in the text.

$\overline{CS}$ **or** δ_0 = the center shift of a Gaussian component in the quadrupole splitting distribution (QSD) of the hyperfine field distribution (HFD) of a given spectral component, given in mm s^{-1} .

$\overline{QS}$ = the center shift of a Gaussian component in the quadrupole splitting distribution (QSD) of the hyperfine field distribution (HFD) of a given spectral component, given in mm s^{-1} .

σ = the Gaussian standard deviation width of a given Gaussian component of a given QSD or HFD.

P = the weight factor (%) for a given Gaussian component in a given QSD or HFD.

H and $\overline{H}$ = the average magnitude of the hyperfine field (expressed as an excited state Zeeman splitting, in Torr (T), in a given HFD of a given sextet spectral component, or all components, respectively.

Δ = the average magnitude of the quadrupole splitting in a given QSD of a given doublet spectral component, given in mm s^{-1}.

$\overline{\epsilon}$ = the average magnitude of the slave distribution of quadrupole shifts (ϵ) associated to a given HFD of a given sextet spectral component, given in mm s^{-1}.

χ^2_ν = the reduced chi-squared value for the fit: chi-squared (χ^2) divided by the number of degrees of freedom (ν). It has an ideal value of 1 for a correct model.

All fits performed using the Voigt-based fitting method of Rancourt and Ping (1991) with the Recoil^{TM} software.

All fitting and calculated parameters are as defined in by Rancourt and Ping (1991).

All $\delta - 1$ couplings between CS and H (or DELTA) were taken to be zero.

All line-1 to line-2 area ratios in all (distributed) elemental doublets were taken to be 1.

All line-2/line-3 and line-1/line-3 area ratios in all (distributed and symmetric) elemental sextets were taken to be 2 and 3, respectively.

All $\epsilon - 1$ couplings between epsilon and H (in a HFD) are taken to be 0.

All Lorentzian half widths at half maximum (HWHM) are set at 0.1425 mm s^{-1} as measured on Fe foil standards on the instrument.

All center shifts ($\overline{CS}$ or δ_0) are given with respect to the CS of metallic Fe at 295K.

Table S6. Mössbauer spectral parameters for soils collected at the end of the last (third) oxic interval, for the redox oscillation treatment **Ox-8** (oxygenation for 8 h). The spectra are presented in Figure S6.

Sample	Phase	Area	$\overline{CS}$ or δ_0	$\overline{\epsilon}$	P	Δ or H	σ	$\overline{QS}$ or $\overline{H}$	χ^2_ν
Ox-72 50K	QSD site 1	25.6(19)	0.380(13)	n/a	100*	0.838(21)	0.281(31)	0.84	0.55
	QSD site 2	13.7(13)	1.101(20)	n/a	100*	3.152(39)	0.258(51)	3.15	
	QSD site 3	10.8(12)	0.858(13)	n/a	100*	1.253(27)	0.05(11)	1.25	
	HFD site 1	36.1(25)	0.483(18)	-0.110(18)	61.5807*	48.55(16)	1.24(28)	45.69	
					38(13)	41.1(31)	6.1(23)		
(BG = 4.8 MC/ch)	HFD site 2	13.7(37)	0.48*	0*	100*	0*	20*	15.96	
Ox-72 35K	QSD site 1	21.0(19)	0.359(17)	n/a	100*	0.845(27)	0.251(41)	0.84	0.62
	QSD site 2	14.1(15)	1.098(21)	n/a	100*	3.177(43)	0.254(56)	3.18	
	QSD site 3	11.8(15)	0.844(18)	n/a	100*	1.238(36)	0.134(63)	1.24	
	HFD site 1	43.7(29)	0.487(19)	-0.123(19)	53.6259*	48.85(18)	1.24(28)	46.1	
					46(10)	42.9(23)	6.2(13)		
(BG = 3.7 MC/ch)	HFD site 2	9.4(43)	0.48*	0*	100*	0*	20*	15.96	
Ox-72 25K	QSD site 1	22.3(17)	0.361(15)	n/a	100*	0.907(25)	0.259(39)	0.91	0.66
	QSD site 2	15.3(13)	1.089(16)	n/a	100*	3.192(33)	0.209(47)	3.19	
	QSD site 3	14.0(14)	0.839(16)	n/a	100*	1.273(33)	0.172(51)	1.27	
	HFD site 1	41.7(24)	0.485(14)	-0.120(14)	62.8113*	49.24(16)	0.99(38)	47.89	
					37(31)	45.6(29)	2.7(18)		
(BG = 5 MC/ch)	HFD site 2	6.6(37)	0.48*	0*	100*	0*	20*	15.96	
Ox-72 13K	QSD site 1	18.2(16)	0.315(19)	n/a	100*	0.885(31)	0.247(46)	0.88	0.66
	QSD site 2	13.0(13)	1.077(17)	n/a	100*	3.219(35)	0.175(56)	3.22	
	QSD site 3	14.0(14)	0.831(16)	n/a	100*	1.293(33)	0.173(53)	1.29	
	HFD site 1	46.9(24)	0.471(11)	-0.106(11)	51.591*	49.63(14)	0.66(30)	48.41	
					48(19)	47.1(12)	2.40(63)		
(BG = 4.4 MC/ch)	HFD site 2	7.8(36)	0.48*	0*	100*	0*	20*	15.96	
Ox-72 5K	QSD site 1	14.0(12)	0.424(13)	n/a	100*	0.745(26)	0.288(45)	0.75	0.71
	QSD site 2	9.18(92)	1.229(13)	n/a	100*	2.894(26)	0.206(44)	2.89	
	QSD site 3	4.62(82)	0.849(26)	n/a	100*	1.159(58)	0.137(83)	1.16	
	HFD site 1	48.3(28)	0.4758(54)	-0.1118(53)	55.7568*	49.514(66)	0.84(12)	48.51	
					44.2(60)	47.25(50)	3.21(30)		
	HFD site 2	17.7(32)	0.48*	0*	100*	0*	20*	15.96	
(BG = 3 MC/ch)	HFD site 3	6.1(37)	1.47(29)	1*	100*	0*	11.5(60)	9.16	

BG = background level, in mega-counts per channel (MC/ch).

Phase = assigned spectral component, as described in the text.

$\overline{CS}$ **or** δ_0 = the center shift of a Gaussian component in the quadrupole splitting distribution (QSD) of the hyperfine field distribution (HFD) of a given spectral component, given in mm s^{-1} .

$\overline{QS}$ = the center shift of a Gaussian component in the quadrupole splitting distribution (QSD) of the hyperfine field distribution (HFD) of a given spectral component, given in mm s^{-1} .

σ = the Gaussian standard deviation width of a given Gaussian component of a given QSD or HFD.

P = the weight factor (%) for a given Gaussian component in a given QSD or HFD.

H and $\overline{H}$ = the average magnitude of the hyperfine field (expressed as an excited state Zeeman splitting, in Torr (T), in a given HFD of a given sextet spectral component, or all components, respectively.

Δ = the average magnitude of the quadrupole splitting in a given QSD of a given doublet spectral component, given in mm s^{-1}.

$\overline{\epsilon}$ = the average magnitude of the slave distribution of quadrupole shifts (ϵ) associated to a given HFD of a given sextet spectral component, given in mm s^{-1}.

χ^2_ν = the reduced chi-squared value for the fit: chi-squared (χ^2) divided by the number of degrees of freedom (ν). It has an ideal value of 1 for a correct model.

All fits performed using the Voigt-based fitting method of Rancourt and Ping (1991) with the Recoil^{TM} software.

All fitting and calculated parameters are as defined in by Rancourt and Ping (1991).

All $\delta - 1$ couplings between CS and H (or DELTA) were taken to be zero.

All line-1 to line-2 area ratios in all (distributed) elemental doublets were taken to be 1.

All line-2/line-3 and line-1/line-3 area ratios in all (distributed and symmetric) elemental sextets were taken to be 2 and 3, respectively.

All $\epsilon - 1$ couplings between epsilon and H (in a HFD) are taken to be 0.

All Lorentzian half widths at half maximum (HWHM) are set at 0.1425 mm s^{-1} as measured on Fe foil standards on the instrument.

All center shifts ($\overline{CS}$ or δ_0) are given with respect to the CS of metallic Fe at 295K.

Table S7. Linear regression of Fe^{II} concentration (mmol kg^{-1}) and cumulative CH_4 (μmol kg^{-1}) with slopes and R^2 for each treatment comparison. See Figure S9.

**Treatment**	**Linear regression (Fe^{II} concentration vs Cumulative CH_4)**	
	**Slope**	**R^2**
All treatments	0.33*	0.18
Pre-conditioning	0.88*	0.77
Ox-72	0.29*	0.62
Ox-24	0.85*	0.65
Ox-8	1.32	0.28

*significant relationship between variables at the 5% probability level.

Supplementary References

(1) Chen, C.; Hall, S. J.; Coward, E.; Thompson, A. Iron-mediated organic matter decomposition in humid soils can counteract protection. *Nature Communications* **2020**, *11* (1), 2255.
(2) Apprill, A.; McNally, S.; Parsons, R.; Weber, L. Minor revision to V4 region SSU rRNA 806R gene primer greatly increases detection of SAR11 bacterioplankton. *Aquatic Microbial Ecology* **2015**, *75* (2), 129-137.
(3) Parada, A. E.; Needham, D. M.; Fuhrman, J. A. Every base matters: assessing small subunit rRNA primers for marine microbiomes with mock communities, time series and global field samples. *Environmental Microbiology* **2016**, *18* (5), 1403-1414.
(4) Illumina. Illumina 16S Metagenomic Sequencing Library Preparation (Illumina Technical Note 15044223). **2013**. Available online: https://support.illumina.com/documents/documentation/chemistry_documentation/16s/16s-metagenomic-libraryprep-guide-15044223-b.pdf (accessed on 5 February 2025).
(5) Caporaso, J. G.; Kuczynski, J.; Stombaugh, J.; Bittinger, K.; Bushman, F. D.; Costello, E. K.; Fierer, N.; Peña, A. G.; Goodrich, J. K.; Gordon, J. I.; et al. QIIME allows analysis of high-throughput community sequencing data. *Nature Methods* **2010**, *7* (5), 335-336.
(6) Yilmaz, P.; Parfrey, L. W.; Yarza, P.; Gerken, J.; Pruesse, E.; Quast, C.; Schweer, T.; Peplies, J.; Ludwig, W.; Glöckner, F. O. The SILVA and "All-species Living Tree Project (LTP)" taxonomic frameworks. *Nucleic Acids Research* **2013**, *42* (D1), D643-D648.
(7) Edgar, R. C. MUSCLE: multiple sequence alignment with high accuracy and high throughput. *Nucleic Acids Research* **2004**, *32* (5), 1792-1797.
(8) McMurdie, P. J.; Holmes, S. phyloseq: An R Package for Reproducible Interactive Analysis and Graphics of Microbiome Census Data. *PLOS ONE* **2013**, *8* (4), e61217.
(9) Love, M. I.; Huber, W.; Anders, S. Moderated estimation of fold change and dispersion for RNA-seq data with DESeq2. *Genome Biology* **2014**, *15* (12), 550.
(10) Martin, M. Cutadapt removes adapter sequences from high-throughput sequencing reads. *EMBnet. journal* **2011**, *17* (1), 10-12.
(11) Callahan, B. J.; McMurdie, P. J.; Rosen, M. J.; Han, A. W.; Johnson, A. J. A.; Holmes, S. P. DADA2: High-resolution sample inference from Illumina amplicon data. *Nature Methods* **2016**, *13* (7), 581-583.
(12) Katoh, K.; Standley, D. M. MAFFT multiple sequence alignment software version 7: improvements in performance and usability. (1537-1719 (Electronic)). From 2013 Apr.
(13) Price, M. N.; Dehal, P. S.; Arkin, A. P. FastTree 2 – Approximately Maximum-Likelihood Trees for Large Alignments. *PloS one* **2010**, *5* (3), e9490-e9490.
(14) Wang, Q.; Cole James, R. Updated RDP taxonomy and RDP Classifier for more accurate taxonomic classification. *Microbiology Resource Announcements* **2024**, *13* (4), e01063-01023.
(15) Barcellos, D.; Cyle, K. T.; Thompson, A. Faster redox fluctuations can lead to higher iron reduction rates in humid forest soils. *Biogeochemistry* **2018**, *137* (3), 367-378.
(16) Reyes, I.; Torrent, J. Citrate-ascorbate as a highly selective extractant for poorly crystalline iron oxides. *Soil Science Society of America Journal* **1997**, *61* (6), 1647-1654.
